# Systematic Characterization of Flavor Profiles and Screening of Potential Key Aroma-Active Components in *Prunus salicina* var. *cordata* cv. ‘Younai’

**DOI:** 10.3390/foods15101787

**Published:** 2026-05-18

**Authors:** Lijuan Fu, Wenjing Liu, Lihua Ren, Xiangxin Lin, Jia Guo, Hao Chen, Faxing Chen, Sun’an Yan

**Affiliations:** 1 Institute of Quality Standards & Testing Technology for Agro-Products, Fujian Academy of Agricultural Sciences/Fujian Key Laboratory of Agro-Products Quality and Safety, Fuzhou 350003, China; fulijuan_f@163.com (L.F.); liuwj163163@163.com (W.L.); rlhlyx123@163.com (L.R.); linxiangxin2008@163.com (X.L.); 15960102868@163.com (J.G.); 2 College of Horticulture, Fujian Agriculture and Forestry University, Fuzhou 350002, China; 3 Fujian Green Food Development Center, Fuzhou 350003, China; chenhao007321@sina.com

**Keywords:** *Prunus salicina* var. *cordata* cv. ‘Younai’, flavor profiles, sugar–acid components, aroma-active compounds, metabolites

## Abstract

*Prunus salicina* var. *cordata* cv. ‘Younai’ is a characteristic stone fruit germplasm resource of Fujian Province, yet its core flavor components remain unclear. This study aimed to comprehensively characterize the flavor chemistry of Younai by determining the contents of sugar–acid components and volatile compounds, combined with untargeted metabolomics analysis. Results showed that fructose was the predominant sweet component, while malic acid was the dominant organic acid in Younai. (Z)-3-Hexen-1-ol, 1-Hexanol and (Z)-3-Hexen-1-yl acetate were screened as potential key aroma-active compounds based on odor activity values, and γ-Decalactone plus Linalool further enriched the fruit aroma hierarchy. Untargeted metabolomics identified 26 saccharides and 21 organic acids in the fruits. Additionally, Younai fruit metabolites were dominated by polyphenols, with flavonols and phenolic acids as the core polyphenol subclasses. This study provides a chemically grounded baseline characterization of Younai flavor. The screened potential aroma-active compounds and metabolite profiles provide a foundation for future sensory validation studies and the development of quality evaluation criteria.

## 1. Introduction

*Prunus salicina* var. *cordata* cv. ‘Younai’ is a unique cultivated variety of Chinese plum endemic to Fujian Province, China, mainly distributed in core producing areas such as eastern and northern Fujian. It is a distinctive fruit tree with both fresh-eating and processing value, featuring crisp tender flesh, a well-balanced sweet–sour taste and fresh fruit aroma [1]. According to the Fujian Provincial Bureau of Statistics, the planting area of Younai has reached 300,000 mu (1 mu ≈ 666.7 m^2^). As a core producing area, Gutian County has a planting area of 62,000 mu for Younai, with an annual output of approximately 30,000 tons and an annual output value exceeding 140 million RMB. Furthermore, “Gutian Younai” has been awarded the National Geographical Indication Certification Trademark. Its products are sold to Hong Kong, Macao and Southeast Asian markets, and an integrated industrial chain covering production, supply, sales and processing has been formed, making it one of the pillar industries for rural revitalization in the region.

Flavor quality is the core factor determining the commercial competitiveness and market potential of Younai [2,3]. Fruit flavor is mainly determined by the combined action of water-soluble sugar–acid taste-active compounds and volatile aroma compounds [3]. Among these, the composition and ratio of soluble sugars and organic acids form the core material basis of sweet–sour flavor [4]. Meanwhile, metabolites provide crucial support for the synthesis and accumulation of flavor compounds, and in-depth analysis of the flavor metabolic mechanisms of Younai fruits offers a theoretical basis for the quality improvement of *Prunus* fruit tree germplasm resources.

Flavor research on stone fruits such as plum, peach and apricot has developed a well-established research system [5,6,7]. In terms of soluble sugar and organic acid components, systematic qualitative and quantitative methods have been established for major sweet compounds (i.e., fructose, glucose, sucrose) and key acidic components (i.e., malic acid, citric acid) [6,8]. For volatile aroma compounds, various aroma components including esters, lactones, alcohols and aldehydes have been identified [5,9], and signature key aroma-active compounds such as hexyl acetate, γ-decalactone and linalool have been screened out via the odor activity value, thus fully constructing the characteristic aroma profiles of stone fruits [5,10]. In addition, combined with metabolomics techniques, core metabolic pathways closely related to flavor compound synthesis have been excavated, such as starch–sucrose metabolism, the tricarboxylic acid cycle and phenylpropane–flavonoid metabolism [11].

Despite the broad understanding of stone-fruit flavor chemistry accumulated for species such as peach, apricot, and plum, systematic flavor characterization of Younai remains notably limited. First, no systematic quantitative analysis has been conducted on core sugar–acid components such as fructose, glucose and malic acid, making it impossible to identify the key factors driving its sweet–sour flavor. Second, there exists a notable gap in flavor research, with no potential aroma-active compounds that actually contribute to its aroma screened out. Third, the metabolic background underlying flavor compound formation remains unclear, which fails to support the demands for cultivar breeding and quality standardization evaluation. To date, no comprehensive study has simultaneously profiled the sugars, organic acids, volatile compounds, and metabolites of Younai fruit across multiple cultivars and production sites. This gap limits both scientific understanding of Younai flavor and the development of quality evaluation criteria for this cultivar.

This study aimed to characterize the flavor profiles of Younai fruits. We used fruits at commercial ripening stage of three main cultivated Younai cultivars as experimental materials. These fruits were sampled from six representative orchards in five core producing areas of Fujian Province. High-performance liquid chromatography (HPLC), headspace solid-phase microextraction–gas chromatography–mass spectrometry (HS-SPME-GC-MS) and ultra-performance liquid chromatography–quadrupole time-of-flight mass spectrometry (UPLC-QTOF-MS) were employed to determine the taste-active components (soluble sugars and organic acids), volatile compounds and metabolic profiles of Younai fruits. These analyses screened the potential key aroma-active compounds and their preliminary associations with metabolite classes, filling the research gap in Younai flavor studies and providing theoretical support for the quality standardization evaluation of Younai.

## 2. Materials and Methods

### 2.1. Experimental Materials and Reagents

#### 2.1.1. Plant Materials

The tested Younai cultivars were Shiban Wannai (SB), Cuiping Younai (CP) and Wanhuangjin (WHJ), with two sampling sites set for each cultivar. The sampling information and corresponding sample codes are listed as follows: SB with two samples, collected from Jianyang Germplasm Resource Bank in Jianyang District, Nanping City, China, (SB-JY, geographic coordinates 27.35° N, 118.02° E) and Jiushan Family Farm in Gutian County, Ningde City, China, (SB-GT, geographic coordinates 26.63° N, 118.71° E). CP with two samples, collected from Caiheli Family Farm in Youxi County, Sanming City, China, (CP-YX, geographic coordinates 26.29° N, 117.72° E) and Zhengzhonghai Family Farm in Gutian County, Ningde City, China, (CP-GT, geographic coordinates 26.68° N, 118.77° E). WHJ with two samples, collected from Yinglian Family Farm in Liancheng County, Longyan City, China, (WHJ-LC, geographic coordinates 25.88° N, 116.72° E) and Liaochunsheng Family Farm in Jian’ou District, Nanping City, China, (WHJ-JO, geographic coordinates 26.82° N, 118.33° E). The quantified data of producing areas, production units and basic fruit traits for each sampling site are detailed in Supplementary Table S1.

All samples were mature fruits with stable commercial properties, and the sampling time of each cultivar was based on the full bloom stage. SB was sampled at 150 d after full bloom (DAFB), CP at 135 DAFB, and WHJ at 165 DAFB. The sampling orchards were under standardized management. For each orchard, three independent trees were selected as biological replicates. From each selected tree, 20 fruits at commercial maturity were randomly collected and pooled to prepare one homogenized sample per tree. Consequently, each orchard yielded three biological replicates (*n* = 3), with each replicate representing an independent pooled fruit batch from a single tree. These three biological replicate samples were analyzed independently for all subsequent chemical analyses. After harvesting, the fruits were immediately placed in a 4 °C constant temperature incubator to eliminate field heat, and then transported to the laboratory rapidly. Upon arrival at the laboratory, the peel and stone were manually removed. The mesocarp was collected, cut into small pieces, snap-frozen in liquid nitrogen, and stored in an ultra-low temperature refrigerator at −80 °C for subsequent index determination.

#### 2.1.2. Reagents

Hydrochloric acid, sodium hydroxide, Folin–Ciocalteu reagent and other reagents were of analytical grade and purchased from China National Pharmaceutical Group Chemical Reagent Co., Ltd., Shanghai, China. Chromatographic grade standard substances (purity ≥ 98.0%), including fructose, sucrose, glucose, sorbitol, malic acid, citric acid, tartaric acid and 3-octanol, were obtained from Sigma-Aldrich (St. Louis, MI, USA). Chromatographic grade solvents such as methanol, acetonitrile and formic acid were purchased from Merck (Darmstadt, Germany) and Fisher Scientific (Waltham, MA, USA), respectively.

### 2.2. Determination of Total Soluble Solids

A PAL-1 handheld refractometer (ATAGO Co., Ltd., Tokyo, Japan) was used to determine the total soluble solids (TSS) content of fruits. Six fruits with uniform maturity and size were selected for each sample, rinsed with distilled water and air-dried. The fruits were pitted, chopped, homogenized and juiced, and the juice was collected for TSS determination after filtration through four layers of gauze.

### 2.3. Determination of Titratable Acid

The stoned plum flesh was homogenized, diluted with water to a constant volume, and then stood still prior to filtration. The obtained supernatant was subjected to neutralization titration with 0.1 mol/L standard sodium hydroxide solution using phenolphthalein as the indicator. The titration end point was recorded when the solution turned pale red and remained unchanged for 30 s. A blank test was conducted simultaneously, and each sample was determined in triplicate. The titratable acid (TA) content was calculated with malic acid (conversion coefficient of 0.067) as the conversion standard.

### 2.4. Determination of Fruit Firmness

A TA.XT Plus texture analyzer (Stable Micro Systems, Godalming, Surrey, UK) was used to determine the firmness of whole Younai fruits in the Texture profile analysis (TPA) mode, with a 36 mm diameter cylindrical probe (P/36) adopted. The test parameters were set as follows: pre-test, test and post-test speeds all at 1.0 mm/s, target deformation at 10%, trigger force at 6 g, and probe interval time at 5 s.

### 2.5. Determination of Sugar Component Contents

The method was adapted from Xiao et al. [6] with minor modifications: Accurately weigh 1.00 g of frozen powder sample, add 10 mL of ultrapure water, vortex-mix thoroughly, and perform ultrasonic extraction for 30 min (200 W, 30 °C), followed by centrifugation at 12,000 r/min for 10 min. The supernatant was collected, the extraction was repeated twice, and all supernatants were combined. The combined supernatant was filtered through a 0.22 μm aqueous filter membrane, and the filtrate was collected for subsequent use.

The Waters E2695 HPLC system (Waters, Milford, MA, USA) equipped with a refractive index detector (RID) and an amino chromatographic column (4.6 mm × 250 mm, 5 μm) was employed. The mobile phase was acetonitrile–ultrapure water (75:25, *v*/*v*) with a flow rate of 1.0 mL/min, column temperature of 30 °C, and injection volume of 20 μL. Qualitative analysis was conducted by comparing the retention times with those of fructose, sorbitol, glucose and other standard substances. Quantitative analysis was performed via the external standard method: standard curves were plotted, and the contents of sugar components were calculated based on peak areas.

### 2.6. Determination of Organic Acid Contents

The method was adapted from Xiao et al. [6] with minor modifications. The sample pretreatment was performed according to the procedure described for sugar component analysis. High-performance liquid chromatography (HPLC, Waters E2695, Waters Corporation, Milford, MA, USA) was employed, equipped with an ultraviolet (UV) detector (detection wavelength: 210 nm) and a C18 chromatographic column (4.6 mm × 250 mm, 5 μm). The mobile phase was 0.05 mol/L potassium dihydrogen phosphate solution (pH 2.5), with a flow rate of 0.8 mL/min, a column temperature of 25 °C and an injection volume of 20 μL. Qualitative analysis was conducted by comparing the retention times with those of standard substances including malic acid, citric acid, tartaric acid and quinic acid. Quantitative analysis was performed via the external standard method: standard curves were plotted, and the organic acid contents were calculated based on peak areas.

### 2.7. Volatile Compounds Analysis

#### 2.7.1. GC-MS/MS Determination

The determination of volatile substances was conducted according to the method described by Zhang et al. [12] with partial modifications: Accurately weigh 1.00 g of Younai fruit sample, grind it in liquid nitrogen, transfer it to a sample vial, add 1 mL of saturated NaCl solution and 10 μL of internal standard (3-octanol, final concentration 11.36 μg/g), and vortex-mix thoroughly. A SPME system (Cat. No.: 5610-5861, Switzerland) was used for volatile aroma extraction, with a 1.1 mm diameter three-phase coated fiber (DVB/CarbonWR/PDMS) paired with a CTC PAL auto-sampler. The SPME fiber was preconditioned as recommended by the manufacturer before the first extraction. The extraction conditions were set as follows: equilibrium at 40 °C for 30 min, followed by SPME fiber extraction for 30 min, then injected into a gas chromatography–tandem mass spectrometry (GC-MS/MS) system.

A Shimadzu GCMS-TQ8050 system (Shimadzu Corporation, Kyoto, Japan) was employed for the determination of volatile compounds, equipped with a DB-5MS capillary column (30 m × 0.25 mm × 0.25 μm). The inlet temperature was set at 250 °C, with splitless injection. High-purity helium (purity ≥ 99.999%) was used as the carrier gas at a flow rate of 1.0 mL/min. The temperature program was as follows: initial temperature 40 °C, held for 3 min; increased to 150 °C at 5 °C/min; and then increased to 250 °C at 10 °C/min, held for 5 min. The ion source was electron impact ionization with an ionization energy of 70 eV, ion source temperature of 230 °C, interface temperature of 280 °C, and solvent delay of 3 min.

#### 2.7.2. Semi-Quantification and Relative Odor Activity Value (rOAV) Calculation

Volatile compounds were tentatively identified via mass spectral matching against the NIST 17.0 database with a similarity index (SI) of no less than 80. For quantification, 3-octanol was added as internal standard at 11.36 ug/g, and all volatile compounds were semi-quantified relative to this internal standard. All contents were quantified as μg/g FW. These data represent relative abundances instead of accurate absolute concentrations.

The relative odor activity value (rOAV) is a well-established method for identifying key flavor compounds in food matrices, which integrates the quantified concentration of each volatile compound with its odor threshold to assess its contribution to the overall aroma profile of the sample. This approach has gained widespread application in recent years to characterize aroma-active compounds across diverse food systems. In general, a compound is considered to make a direct contribution to the characteristic flavor of the sample if its calculated rOAV is ≥1. In this study, rOAV values were determined following previously described protocols [13,14,15] using the formula

$$r{OAV}_{i}=\frac{C_{i}}{T_{i}}$$

where *rOAV_i_* is the relative odor activity value of compound *i*, *C_i_* is the concentration of compound *i* (μg/g FW), and *T_i_* is its corresponding odor threshold (μg/g). The odor threshold data were cited from Ref. [16]. A compound is generally considered to make a direct contribution to the characteristic aroma of the sample if its rOAV is ≥1.

It should be noted that this study employed chemical quantification and rOAV screening as a foundational approach to identify potential aroma-active compounds. While an rOAV above 1 is conventionally interpreted as indicating potential aroma relevance, definitive confirmation of flavor-driving components would require complementary sensory validation methods such as gas chromatography–olfactometry (GC-O), descriptive sensory analysis, or aroma recombination studies, which were not performed in the present work.

### 2.8. Determination of Metabolites

The determination of metabolites in Younai was performed using the method established in our laboratory. Sample Pretreatment: Younai samples were lyophilized and ground into powder. Then, we accurately weighed 0.100 g of powder into a 15 mL centrifuge tube, added 10 mL of 80% methanol–water, shook for extraction for 15 min, and centrifuged at 10,000 rpm at 4 °C. Finally, the supernatant was filtered through a 0.22 μm organic filter membrane and transferred to a sample vial.

Instrumental analysis was conducted on a SCIEX X500R UPLC-QTOF-MS system (SCIEX, Marlborough, MA, USA). The liquid chromatography conditions were as follows: HSS T3 chromatographic column (1.8 μm, 2.1 × 100 mm). Mobile phase A: 0.01% formic acid water; mobile phase B: acetonitrile. Flow rate: 0.4 mL/min. Column temperature: 40 °C. Injection volume: 1 μL. Gradient elution program: 1.00 min 95%A/5%B, 1.10 min 60%A/40%B, 16.00 min 50%A/50%B, 24.00 min 10%A/90%B, 28.00 min hold 10%A/90%B, 28.10 min return to 95%A/5%B, 30.00 min end.

The mass spectrometry conditions were: ESI ion source (positive/negative ion mode). Acquisition mode: TOF MS-MS/MS. TOF MS mass-to-charge ratio range: 100–1300 Da. TOF MS/MS mass-to-charge ratio range: 50–1300 Da. CDS and DBS were enabled. Ion source parameters included: ESI voltage 5500 V (positive)/−4500 V (negative), curtain gas 30 psi, nebulizer gas GAS2 55 psi, GAS1 50 psi, source temperature 550 °C, declustering potential 60 V (positive)/−60 V (negative), collision energy 35 ± 15 V (positive)/−35 ± 15 V (negative).

Quality control (QC) was ensured by preparing a pooled QC sample from equal aliquots of all biological samples. This QC sample was injected every 10 samples throughout the run sequence. QC clustering was assessed by principal component analysis (PCA). QC samples clustered tightly together and distinctly from biological samples, confirming analytical stability. Only metabolites with a relative standard deviation (RSD) < 30% across QC injections were retained for downstream analysis. Data normalization was performed using total ion current (TIC) normalization. Missing values (occurring when a metabolite was detected below the limit of detection in some samples) were handled as follows: metabolites with missing values in >50% of samples within any group were excluded. Remaining missing values were imputed with half the minimum positive value in the dataset. Substance identification was conducted through the SCIEX OS database and the One-Map platform cloud database (using MExplorer Ultimate software, me.5omics.com, format: accessed on 1 August 2025), with a primary mass deviation <5 ppm. QC samples exhibited tight clustering in the PCA score plot (Supplementary Figure S1), verifying the stability and reproducibility of the metabolomics analysis.

### 2.9. Data Analysis Methods

A total of six fruit samples of Younai were harvested at the commercial full-ripening stage in this study, with three biological replicates set for each sample. All samples were unique cultivar-orchard combinations, specifically coded as SB-JY, SB-GT, CP-YX, CP-GT, WHJ-JO, and WHJ-LC, covering three major cultivated cultivars and five core production orchards of Younai. Statistical analysis was performed using SPSS 25.0 software (IBM Corp., Armonk, NY, USA). Data were analyzed by one-way ANOVA, with independent Younai sample type set as the single factor (six levels). Post hoc multiple comparisons were conducted using Tukey’s honestly significant difference (HSD) test to analyze the differences in the contents of sugars, organic acids and other flavor-related compounds among different samples. *p* < 0.05 was considered statistically significant. Result charts were drawn using OriginPro 2021, MetWare Cloud Platform (https://cloud.metware.cn, format: accessed on 1 December 2025) and Omicshare (https://www.omicshare.com/, format: accessed on 1 December 2025).

## 3. Results

### 3.1. Analysis of Morphological Characteristics and Basic Sugar, Acid and Texture Indicators of Younai Fruits

**Figure 1:**
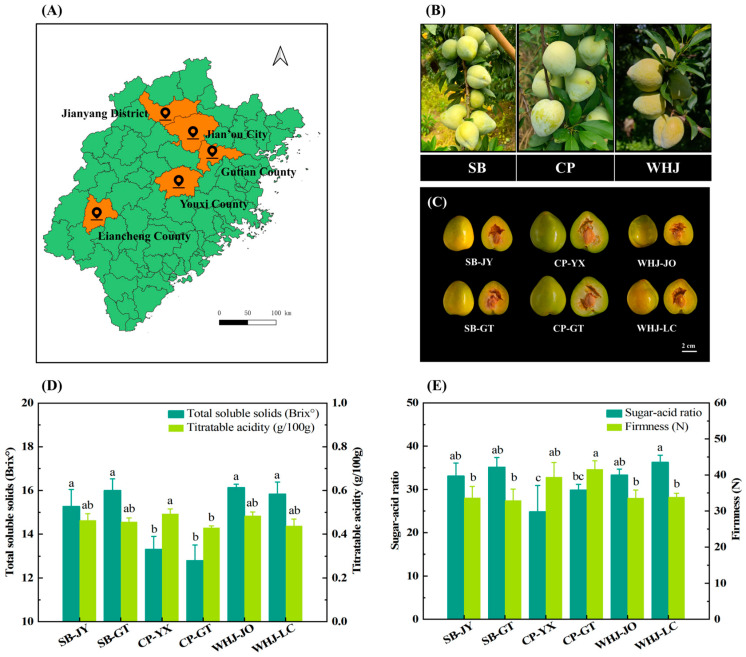
Analysis of morphological characteristics and basic sugar, acid and firmness indicators of Younai fruits. (**A**) Sampling sites of five major core producing areas in Fujian Province; (**B**) fruiting morphology of three main cultivated Younai cultivars; (**C**) cross-sectional morphology of Younai fruits from different producing areas (scale bar = 2 cm); (**D**) contents of total soluble solids (TSS, °Brix) and titratable acid (TA, calculated as malic acid, g/100 g); (**E**) sugar–acid ratio and fruit firmness (N). Data are presented as mean values ± standard deviation (*n* = 3). Different letters indicate significant differences (*p* < 0.05). SB means Shiban Wannai, CP means Cuiping Younai, WHJ means Wanhuangjin. SB-JY: Shiban Wannai from Jianyang District; SB-GT: Shiban Wannai from Gutian County; CP-YX: Cuiping Younai from Youxi County; CP-GT: Cuiping Younai from Gutian County; WHJ-JO: Wanhuangjin from Jian’ou City; WHJ-LC: Wanhuangjin from Liancheng County. Other figure annotations are the same as above.

As shown in Figure 1A, the sampling sites were Jianyang District, Gutian County, Youxi County, Liancheng County and Jian’ou City in Fujian Province. According to the data from Fujian Provincial Bureau of Statistics, these areas are all core producing areas of Younai, accounting for more than 80% of the total planting area of Younai in the province. Figure 1B shows the fruiting morphology of three main Younai cultivars. All three cultivars exhibited a cluster-bearing fruiting habit, with peach-shaped fruits and a thin waxy bloom on the surface. Figure 1C shows the cross-sectional morphology of Younai fruits from different producing areas. Among them, CP was light green, while SB and WHJ were light yellowish-green. Combined with the data in Supplementary Table S1, the longitudinal diameter of CP fruits (61.00~62.01 mm) was significantly larger than that of SB (55.48~55.75 mm) and WHJ (55.61~55.64 mm). The single fruit weight of CP was 118.52~124.22 g, while those of SB and WHJ were both between 96~103 g. Although there were differences in fruit size and peel color among the three cultivars, they all maintained the core morphological characteristics of Younai, reflecting the stable germplasm characteristics of the main cultivated Younai cultivars in Fujian Province.

The sweet and sour flavor of Younai fruits is their core edible quality characteristic. Figure 1D shows the TSS and TA contents of Younai samples. The results showed that the TSS content ranged from 12.80 to 16.13 °Brix overall. Among them, the TSS contents of SB and WHJ (15.27~16.13 °Brix) were significantly higher than that of CP (12.80~13.30 °Brix). The TA content fluctuated slightly overall, stably ranging from 0.43 to 0.49 g/100 g. Figure 1E shows the sugar–acid ratio and firmness characteristics of Younai fruits. The sugar–acid ratio is a core indicator reflecting the sweet and sour palatability of fruits. The sugar–acid ratio of Younai samples ranged from 24.80 to 36.25. Among them, the sugar–acid ratios of SB and WHJ samples were relatively high (33.08~36.25), while that of CP was relatively low (24.80~29.82). These differences among cultivars enrich the flavor types of Younai and meet different consumer demands. In terms of fruit firmness, the firmness of CP-GT (38.89~43.21 N) was significantly higher than that of SB (32.90~33.89 N) and WHJ (33.12~33.57 N), and there was no significant difference among different producing areas of the same cultivar.

### 3.2. Analysis of Sugar and Acid Components in Younai Fruits

**Figure 2:**
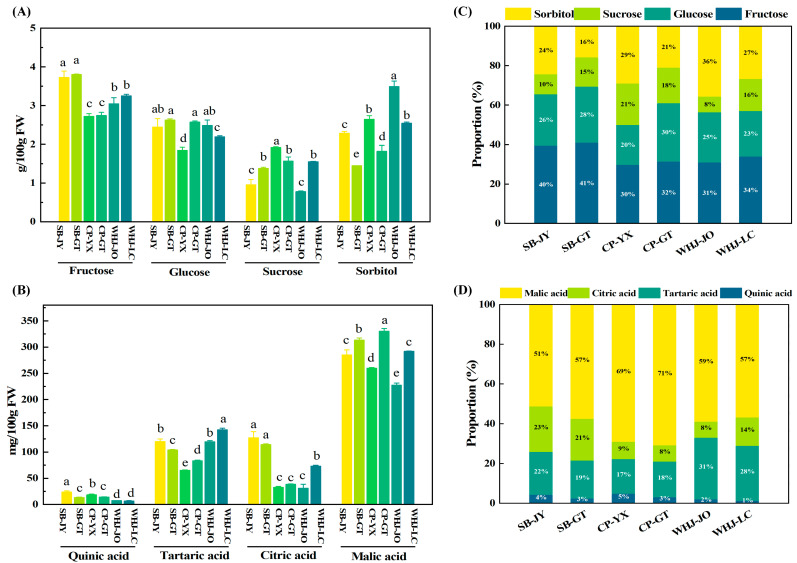
Analysis of sugar and acid components in Younai fruits. (**A**) Contents of major sugar components (fructose, glucose, sucrose, sorbitol) (g/100 g); (**B**) contents of major organic acid components (quinic acid, tartaric acid, citric acid, malic acid) (mg/100 g); (**C**) proportions of major sugar components; (**D**) proportions of major organic acid components. Data are presented as mean values ± standard deviation (*n* = 3); Different letters indicate significant differences (*p* < 0.05).

To clarify the material basis of core flavor, the identification and content analysis of soluble sugars and organic acids were further carried out. As shown in Figure 2A, the contents of soluble sugar components showed a stable order of fructose > glucose and sorbitol > sucrose in all samples. Fructose was the most dominant soluble sugar, with a content ranging from 2.73 to 3.81 g/100 g. Glucose (1.84~2.63 g/100 g) and sorbitol (1.45~3.49 g/100 g) followed, while sucrose content (0.78~1.92 g/100 g) was relatively low. From the perspective of flavor chemistry, the sweetness coefficient of fructose (1.73) was significantly higher than that of sucrose (1.0), glucose (0.74) and sorbitol (0.6) [6]. As a characteristic polyol of *Prunus* fruits [17], sorbitol not only provides a mild sweet contribution, but also improves the moist taste of fruits and effectively relieves the sweetness cloying caused by high fructose content. This flavor modulation characteristic mediated by characteristic polyols is also an important flavor feature that distinguishes Younai from other stone fruits such as peaches and pome fruits such as apples [18].

Figure 2B presents the contents of core organic acid components in Younai fruits. The average contents of organic acids in the samples showed a stable order of malic acid > tartaric acid and citric acid > quinic acid. Malic acid was the predominant organic acid, with a content ranging from 227.35 to 330.59 mg/100 g, followed by tartaric acid (65.40~142.68 mg/100 g). Citric acid and quinic acid were present at relatively low levels, with contents of 31.22~127.63 mg/100 g and 6.68~24.10 mg/100 g, respectively. As a characteristic organic acid of stone fruits, malic acid has a mild, persistent and non-irritating sour taste [19], and it could generate a flavor synergy with the fresh sweet taste of fructose, which serves as the core material basis for the coordinated sweet–sour taste. Tartaric acid enriches the sour taste layers and effectively compensates for the monotony of sour taste induced by malic acid alone [20]. Citric acid and quinic acid further modulate the sour taste balance of the fruits [21].

### 3.3. Analysis of the Proportion of Sugar and Acid Components in Younai Fruits

Figure 2C shows the proportion characteristics of main sugar components in Younai fruits. Fructose accounted for the highest proportion (more than 30%), being the primary contributor to the sweetness of Younai. Sorbitol and glucose followed in proportion, while sucrose had the lowest proportion. Even though there were cultivar differences in the absolute content of sugar components among different samples, their proportion pattern remained stable, which suggested the high stability of Younai’s sweet flavor at the component structure level. In this study, the sugar composition pattern, with fructose as the main component, glucose and sorbitol as supplements, and sucrose as a minor component, is the core characteristic of Younai germplasm and not significantly affected by the producing area environment.

Figure 2D shows the proportion characteristics of major organic acid components in Younai fruits. The results indicated that malic acid was absolutely dominant in all samples, accounting for more than 50% of the total organic acids. Tartaric acid (17~31%) and citric acid (8~23%) were the next most abundant in proportion, while quinic acid accounted for less than 5%. In this study, consistent with the proportion pattern of sugars, the proportion pattern of organic acids in Younai was conserved across different cultivars and producing areas. This indicated that the organic acid composition pattern dominated by malic acid and supplemented by tartaric acid was the core determinant of the sour flavor of Younai fruits, supporting the germplasm dominance of the sweet–sour flavor in Younai.

Based on the above findings, Younai fruits have established a stable material basis for the sweet–sour flavor that is centered on fructose-malic acid and co-regulated by other soluble sugars and organic acids. This pattern exhibited conservation across the six sample groups (SB-JY, SB-GT, CP-YX, CP-GT, WHJ-JO, and WHJ-LC) from five producing areas selected in this experiment. These results characterize the sugar and organic acid composition of Younai fruit. The perceived balance of sweetness and sourness would require sensory validation. Although environmental factors of producing areas (such as climate, soil and cultivation management practices) exerted a significant effect on the absolute contents of sugar and acid components in Younai fruits, these factors did not alter its core sugar–acid composition pattern.

### 3.4. Composition and Content Distribution of Volatile Compounds in Younai Fruits

Volatile compounds are key components of Younai fruit flavor, which, together with sweet and sour tastes, determine the edible quality. After analysis by headspace solid-phase microextraction coupled with HS-SPME-GC-MS, a total of 28 volatile organic compounds (VOCs) were identified by excluding noise components with a detection rate lower than 30% (Table 1). The principal component analysis (PCA) score plot for volatile compounds is provided in Supplementary Figure S1.

**Figure 3:**
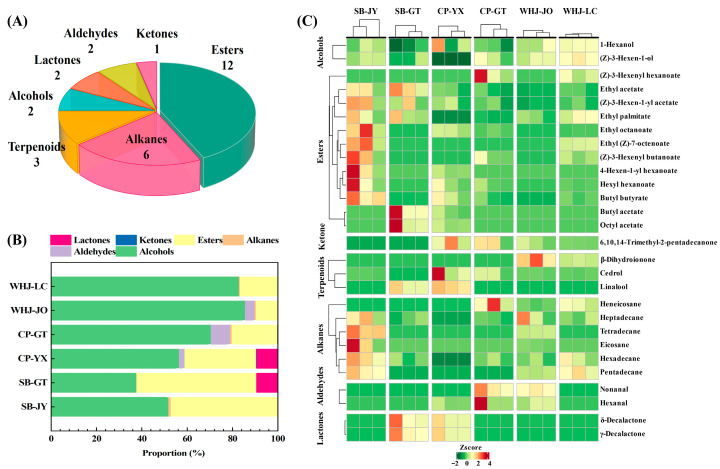
Composition, content distribution and cluster analysis of volatile compounds. (**A**) Pie chart showing the classification of 28 volatile compounds; (**B**) stacked bar chart of volatile compound contents in different groups; (**C**) heatmap of cluster analysis based on volatile compound contents.

These VOCs were classified into seven categories (alcohols, aldehydes, alkanes, esters, ketones, lactones, terpenes) based on their chemical structures. In terms of the number of compounds (Figure 3A), esters were the most abundant with 12 types, accounting for 42.86%; alkanes followed with six types (21.43%). There were three types of alcohols and terpenes each, two types of aldehydes and lactones each, and only one type of ketones. In terms of content proportion (Figure 3B), alcohols were the dominant volatile category with the highest total content, ranging from 33.14 to 75.80 μg/g with an average of 51.25 μg/g. Esters ranked second, with a total content of 7.20–52.02 μg/g and an average of 25.90 μg/g. The contents of alkanes, aldehydes, terpenes, lactones and ketones decreased in turn, with ketones having the lowest content and only being detected in some samples.

Figure 3C presents the differences in the contents of volatile compounds among samples. Ester monomers (ethyl acetate, (Z)-3-hexen-1-yl acetate, ethyl palmitate) were highly accumulated in SB-JY and SB-GT, consistent with the overall trend of prominent ester contents in SB samples shown in Figure 3B. Alcohol monomers (1-hexanol, (Z)-3-hexen-1-ol) were highly accumulated in WHJ-JO and WHJ-LC, matching the high alcohol content trend of WHJ samples in Figure 3B. Alcohols and esters in the highly accumulated volatile compound clusters are the main potential contributors to Younai flavor, and can be regarded as key targets for preliminary screening.

**Figure 4:**
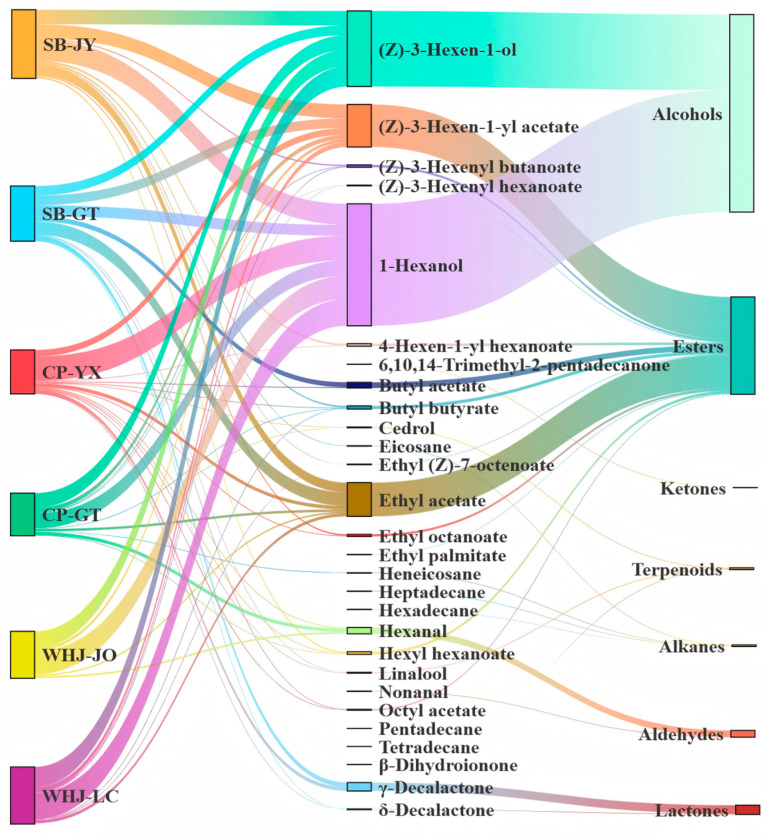
Sankey diagram of flux correlation between volatile aroma compound categories and sample groups in Younai fruit.

The Sankey diagram (Figure 4) of volatile compounds intuitively reflects the corresponding relationship between aroma categories and their contents through the distribution of material flow intensity. The results showed that alcohols had a strong material flow correlation with all sample groups, being the only aroma category with high content detected in all samples and wide correlation, further confirming that alcohols are the common core aroma category of Younai fruits. The correlation between esters and SB-JY and SB-GT was significantly higher than that with CP and WHJ. Aldehydes formed weak correlations with CP-YX, CP-GT and WHJ-JO. Lactones had a trace material flow correlation with SB-GT and CP-YX. Terpenes showed weak and uniform material flow distribution with all sample groups, while alkanes and ketones had the lowest correlation with samples, with scattered material flow and no obvious regularity.

From the perspective of material distribution, the correlation characteristics of the Sankey diagram further explain the rich layers of Younai aroma with “common basis + specific expression”. The broad-spectrum and strong correlation of alcohols makes them the basic aroma background of all Younai samples, while esters, aldehydes and lactones add more flavor layers.

### 3.5. Preliminary Screening of Potential Odor-Active Compounds in Younai Fruits

Studies have shown that the aroma is closely related to the threshold concentration of volatile compounds, which is usually evaluated by relative OAVs [16]. In this study, OAV > 1 was used as the standard to screen the key potential odor-active compounds in Younai fruits. The results are shown in Table 2.

In terms of categories, alcohols and esters were the core categories of potential odor-active compounds, with rOAVs > 1 in all samples, making them the main potential contributors to Younai aroma. Among alcohols, (Z)-3-hexen-1-ol and 1-hexanol were the main content contributors, and their grassy and green leafy flavor characteristics made them the core potential active compounds of the basic aroma of Younai. Among esters, (Z)-3-hexen-1-yl acetate, (Z)-3-hexenyl butanoate, (Z)-3-hexenyl hexanoate and other ester compounds had rOAVs > 1 in all detected samples; most of these compounds had fruity and sweet flavor characteristics, serving as the core potential active compounds of the fruity flavor of Younai. Aldehydes, lactones and terpenes were potential odor-active compounds, which were only detected in some samples with rOAVs > 1. Among aldehydes, hexanal and nonanal added fatty and green leafy flavor layers to Younai. Among lactones, γ-decalactone and δ-decalactone mainly exhibited peach and creamy flavors. Terpenes including linalool and β-dihydroionone exhibit floral and sweet fruity characteristics, and these compounds, based on their rOAV values, potentially contributed to Younai aroma as important regulatory compounds for its aroma layers. Ketones and alkanes had high thresholds, with rOAVs ≤ 1 in all samples, so they could be excluded from the candidates for core odor-active compounds of Younai.

From the perspective of flavor chemistry, the composition of aroma compounds in Younai is consistent with the aroma commonalities of *Prunus* stone fruits [22,23]. As two core aroma components, alcohols and esters together form the main framework of the basic aroma profile of Younai. Alcohols, typical characteristic substances with grassy and green leafy aromas, are the signature aroma during the ripening process of stone fruits [24]. Esters exhibit fruity and sweet aromas [25]. Meanwhile, aldehydes, alcohols, terpenes and lactones add richer layers to the aroma characteristics [24]. The above results define the core target range for the subsequent screening of potential odor-active compounds.

### 3.6. Analysis of the Core Metabolic Basis of Younai Fruit Flavor

**Figure 5:**
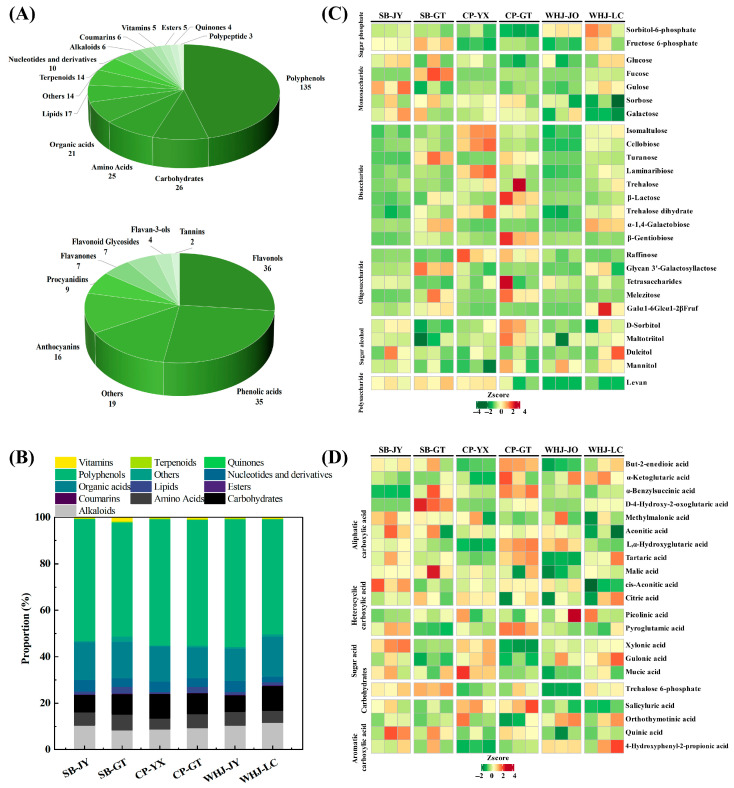
Analysis of metabolite category composition and soluble sugar, organic acid metabolites in Younai fruits. (**A**) Overall category composition of metabolites and composition of polyphenol subcategories in Younai fruits; (**B**) stacked chart of relative peak area proportions of different metabolite categories; (**C**) hierarchical cluster heatmap of soluble sugar metabolite subcategories; (**D**) hierarchical cluster heatmap of organic acid metabolite subcategories in Younai fruits.

The untargeted metabolomics analysis identified 293 metabolites across six major classes, providing a preliminary chemical fingerprint of Younai fruit. The PCA results for metabolites, including QC sample clustering verification, are displayed in Supplementary Figure S2. The 293 metabolites could be divided into 13 major categories based on their chemical structures and functions, clarifying the overall composition characteristics of metabolites in Younai fruits (Figure 5A). Among them, 135 were polyphenols, accounting for 46.06% of the total identified metabolites, making them the largest metabolite category in Younai fruits, followed by sugars (26 species), amino acids (25 species) and organic acids (21 species). These three categories accounted for 25.60% of the total metabolites, and the proportions of other categories were all less than 10%.

Polyphenols could be divided into nine subcategories, among which flavonols (36 species) and phenolic acids (35 species) were the two most abundant subcategories, accounting for 52.59% of the total polyphenols. Followed by anthocyanins (16 species) and proanthocyanidins (nine species), the number of other polyphenol subcategories was small, accounting for less than 20% in total. Flavonols, phenolic acids and proanthocyanidins are the three major polyphenol subcategories closely related to Younai flavor. Studies have shown that flavonols and phenolic acids are not only the main regulatory substances for the astringency of fruits [26], but also can serve as aroma precursors to generate volatile aroma compounds such as aldehydes and alcohols through degradation reactions, participating in the formation of Younai’s fruity and grassy aromas [24]. Proanthocyanidins mainly contribute to the slight astringency of Younai and enhance the flavor layering [27].

Focusing on the core flavor components, further analysis of the 26 soluble sugars and 21 organic acids was performed, with the results shown in Figure 5B. The soluble sugar components encompassed disaccharides, monosaccharides, oligosaccharides, sugar alcohols, sugar phosphates, and polysaccharides, and the contents of monosaccharides varied among different samples. Isomaltulose, raffinose, etc., could impart a mellow flavor to the fruits, while characteristic *Prunus* sugar alcohols such as sorbitol and mannitol were consistently present in all samples. Previous studies have confirmed that these sugar alcohols are characteristic flavor-contributing components of *Prunus* fruits, endowing them with a cool and sweet aftertaste [17].

The organic acid components, as shown in Figure 5C, were identified to belong to categories such as aliphatic carboxylic acids, heterocyclic carboxylic acids, sugar acids, and aromatic carboxylic acids. Among them, aliphatic carboxylic acids such as malic acid, citric acid, and tartaric acid served as the core source of acidity in stone fruits [6], as well as the primary contributor to the acidity of Younai fruits. Other organic acid components, including sugar acids and aromatic carboxylic acids, further modulated the fruit flavor and enhanced the flavor layers, and, in synergy with antioxidant substances, improved the nutritional quality of the fruits [28].

### 3.7. Correlation Analysis of Fruit Quality, Flavor and Volatile Compound Contents in Younai Fruits

**Figure 6:**
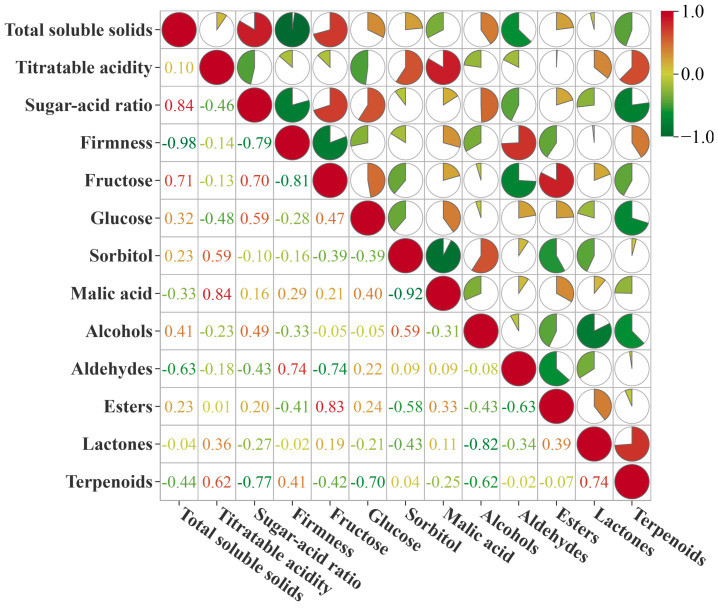
Heatmap of correlation analysis of core flavor compounds in Younai fruits. The color depth in the heatmap represents the magnitude of the correlation coefficient. Red indicates a positive correlation, while green indicates a negative correlation, and the numbers represent the specific correlation coefficient values. Pearson correlation coefficients and raw (uncorrected) *p*-values are reported. The analysis was performed using the MetWare Cloud Platform, which does not apply built-in multiple testing correction. Readers are advised to interpret correlations with *p*-values near the threshold with appropriate caution.

To clarify the synergistic/antagonistic regulatory relationships between core quality indicators, flavor components and volatile compounds in the formation of Younai fruit quality, Pearson correlation analysis was performed on 13 key indicators, with the results shown in Figure 6 (Supplementary Tables S3 and S4). Fruit firmness was extremely significantly negatively correlated with TSS (r = −0.98, *p* = 0.0007), which is consistent with the common ripening rule of stone fruits [29]. Specifically, during fruit ripening and softening, cell wall structure degrades, while photosynthetic products are continuously transported to fruits and converted into soluble sugars, promoting TSS accumulation. TA was significantly positively correlated with malic acid (r = 0.84, *p* = 0.04), confirming that malic acid is the main contributor to the total acidity of Younai fruits.

## 4. Discussion

The above results suggest that Younai has established a basic flavor framework centered on fructose and malic acid taste and combined with characteristic volatile compounds, with the basic skeleton characterized by “fructose-sorbitol-glucose-malic acid-(Z)-3-hexen-1-ol-(Z)-3-hexen-1-yl acetate”, and polyphenols (flavonols and phenolic acids) serving as the core polyphenol metabolites. In the 293 metabolites detected in this study, polyphenol metabolites accounted for a dominant proportion. These polyphenol substances provide Younai with astringent, bitter and antioxidant characteristics, while their degradation products also serve as precursors of aromatic compounds [29]. For example, phenolic acids such as chlorogenic acid and gallic acid can generate aromatic aldehydes through oxidative degradation, while flavonols such as quercetin can release aromatic components during the Maillard reaction [30,31]. This metabolic link between polyphenols and volatile compounds further enriches the flavor diversity and complexity of Younai.

To comprehensively elucidate the flavor formation characteristics of Younai, this study systematically compared its sugar–acid components and volatile aroma profiles with published data from other *Prunus* cultivars and related stone fruit species. For organic acids, malic acid was the absolutely dominant component in Younai, highly consistent with major Japanese plum cultivars including ‘Angeleno’ and ‘Black Amber’ [32]. This aligns with the core organic acid metabolic pattern of *Prunus* fruits, further validating the reliability of our results. Significant cultivar-specific differences were observed in soluble sugars. Fructose was the predominant sugar in Younai, whereas Xiao et al. (2024) [6] reported glucose as the dominant sugar across 86 plum cultivars. This discrepancy may mainly stem from the unique genotype of Younai, combined with effects of harvest maturity, local climate and soil conditions. For volatiles, C6 alcohols and esters were the basal aroma components of Younai, matching the common “fresh fruity” trait of *Prunus* fruits. γ-decalactone and linalool were identified as potential core contributors to Younai’s unique volatile profile, distinguishing it from other plums [32,33]. This trait may be closely related to abundant carotenoids (key precursors for terpenoid and lactone aroma biosynthesis) in Younai’s yellow flesh [23,34], as well as cultivar-specific expression of key aroma biosynthesis genes.

In this study, core polyphenol metabolites, flavonols and phenolic acids, accounted for a dominant proportion of the 293 metabolites. It has been reported in other plant systems that certain phenolic compounds may serve as potential precursors for volatile aldehydes and alcohols through enzymatic and non-enzymatic degradation reactions during fruit ripening and processing [35]. However, it is important to emphasize that the present study did not perform direct precursor-product analysis or pathway-level investigation to establish such links in Younai specifically. The relationship between polyphenol composition and volatile formation in this cultivar therefore remains a hypothesis. Further studies could combine metabolomics with transcriptomics to trace the origin and transformation of polyphenol metabolites in Younai, providing a more comprehensive theoretical basis for its flavor formation mechanism.

It is important to acknowledge several limitations of the present study. First, although we employed rOAV calculations to prioritize potential aroma-active compounds, direct sensory validation—such as descriptive sensory panel evaluation, GC-O, or aroma recombination and omission tests—was not performed. Therefore, the identification of key flavor-driving components in this study represents a chemically grounded screening rather than a sensory-confirmed determination. Second, the metabolomics analysis was primarily descriptive. Pathway enrichment analysis, metabolite–metabolite network analysis, and transcriptomic integration were beyond the scope of this work but would be necessary to establish mechanistic links between metabolite classes and flavor chemistry. Third, the volatile quantification method was semi-quantitative relative to an internal standard rather than fully validated absolute quantification using authentic standard curves for all compounds. Additionally, systematic climate, irrigation and soil matrix data were not collected in this study, which precludes the quantitative analysis of environmental effects on Younai flavor. Meanwhile, our nested sampling design (SB-JY, SB-GT, CP-YX, CP-GT, WHJ-JO, WHJ-LC) prevents clear disentanglement of cultivar and production site effects, further hindering the determination of each factor’s specific contribution to Younai flavor. We also acknowledge differential harvest dates among cultivars as a potential confounding factor, and we plan to conduct sampling with multiple harvest dates per cultivar in future research to clarify temporal effects on flavor chemistry.

In summary, this study provides a useful baseline dataset for the chemical composition of Younai fruit flavor. These data contribute to the understanding of this regionally important cultivar and may serve as a foundation for future studies incorporating sensory validation, transcriptomic analysis, and expanded sampling. The development of a quality standardization evaluation system for Younai will require integration of the present chemical data with direct sensory measurements and broader cultivar–site–season coverage.

## 5. Conclusions

This study provides a comprehensive chemical characterization of flavor-related components in *Prunus salicina* var. cordata cv. ‘Younai’. The results indicate that fructose and malic acid are the predominant sugar and organic acid, respectively, and that C6 alcohols and esters—notably (Z)-3-hexen-1-ol, 1-hexanol, and (Z)-3-hexen-1-yl acetate—are the major volatile compounds. Based on odor activity values, γ-decalactone and linalool were additionally screened as potential contributors to the characteristic aroma of Younai. Untargeted metabolomics identified 293 metabolites, with flavonols and phenolic acids representing the most abundant classes, providing a preliminary chemical fingerprint of this cultivar. This study provided a theoretical basis for the construction of a quality standardization evaluation system.

## Figures and Tables

**Table 1:** Categories and contents of 28 volatile compounds in various Younai fruit samples.

Compound Name	SB-JY	SB-GT	CP-YX	CP-GT	WHJ-JO	WHJ-LC
	μg/g, FW					
(Z)-3-Hexen-1-ol	23.34 ± 3.19	15.39 ± 7.26	—	23.50 ± 8.24	26.71 ± 3.36	32.55 ± 1.99
1-Hexanol	33.78 ± 5.70	17.75 ± 7.49	39.07 ± 19.99	24.61 ± 7.25	37.52 ± 5.77	43.25 ± 0.70
Hexanal	—	—	1.67 ± 0.92	5.59 ± 6.53	3.00 ± 1.03	—
Nonanal	—	—	—	0.24 ± 0.09	0.19 ± 0.02	—
Eicosane	0.70 ± 0.67	—	—	—	0.05 ± 0.03	—
Heneicosane	—	—	—	0.35 ± 0.23	0.05 ± 0.02	0.21 ± 0.05
Heptadecane	0.17 ± 0.08	0.05 ± 0.03	—	0.05 ± 0.01	0.15 ± 0.12	0.08 ± 0.03
Hexadecane	0.15 ± 0.03	0.06 ± 0.02	—	0.06 ± 0.02	0.06 ± 0.02	0.10 ± 0.03
Pentadecane	0.07 ± 0.02	—	—	—	0.04 ± 0.00	0.06 ± 0.01
Tetradecane	0.07 ± 0.01	—	—	—	0.03 ± 0.00	—
(Z)-3-Hexen-1-yl acetate	22.37 ± 9.53	15.42 ± 8.35	10.71 ± 4.57	7.33 ± 3.85	4.83 ± 1.34	7.71 ± 2.14
(Z)-3-Hexenyl butanoate	2.25 ± 1.65	—	—	0.75 ± 0.68	—	0.73 ± 0.29
(Z)-3-Hexenyl hexanoate	—	—	—	0.74 ± 0.70	—	0.39 ± 0.15
4-Hexen-1-yl hexanoate	3.31 ± 2.91	—	0.74 ± 0.37	—	—	—
Butyl acetate	—	7.94 ± 5.71	1.18 ± 1.46	—	—	—
Butyl butyrate	2.74 ± 0.82	0.17 ± 0.01	1.32 ± 0.73	0.35 ± 0.35	—	0.49 ± 0.16
Ethyl (Z)-7-octenoate	0.62 ± 0.35	—	—	—	—	0.30 ± 0.02
Ethyl acetate	15.78 ± 7.08	21.66 ± 7.36	5.62 ± 3.00	3.84 ± 3.23	2.29 ± 0.62	5.07 ± 1.73
Ethyl octanoate	2.26 ± 1.42	—	0.92 ± 0.24	0.23 ± 0.11	—	0.13 ± 0.02
Ethyl palmitate	0.14 ± 0.07	0.13 ± 0.06	—	0.04 ± 0.01	0.07 ± 0.03	0.14 ± 0.02
Hexyl hexanoate	2.56 ± 2.43	—	1.01 ± 1.00	0.43 ± 0.47	—	0.20 ± 0.08
Octyl acetate	—	1.15 ± 0.98	0.31 ± 0.15	—	—	—
6,10,14-Trimethyl-2-pentadecanone	—	—	0.13 ± 0.07	0.11 ± 0.07	0.07 ± 0.03	0.05 ± 0.00
γ-Decalactone	—	7.60 ± 2.99	5.95 ± 1.91	—	—	—
δ-Decalactone	—	0.70 ± 0.35	0.53 ± 0.16	—	—	—
Cedrol	0.09 ± 0.01	—	0.67 ± 0.56	0.28 ± 0.18	0.12 ± 0.01	0.10 ± 0.01
Linalool	—	0.60 ± 0.20	0.80 ± 0.10	—	—	—
β-Dihydroionone	—	—	—	—	0.20 ± 0.07	0.08 ± 0.01

Note: Volatile concentrations represent semi-quantitative estimates relative to an internal standard (3-octanol, 11.36 ug/g) and are expressed as ug/g fresh weight.

**Table 2:** Relative odor aroma activity values (rOAV) of volatile compounds in Younai fruits.

Volatile Compounds	Aroma Thresholds (μg/g)	SB-JY	SB-GT	CP-YX	CP-GT	WHJ-JO	WHJ-LC
		Relative Odor Activity Values (rOAV)					
(Z)-3-Hexen-1-ol	0.05	466.75	307.83	—	470.01	534.29	650.99
1-Hexanol	0.25	135.11	71	156.3	98.43	150.09	172.98
Hexanal	0.011	—	—	151.55	508.37	273.03	—
Nonanal	0.01	—	—	—	23.9	19.13	—
(Z)-3-Hexen-1-yl acetate	0.02	1118.29	770.93	535.54	366.32	241.64	385.68
(Z)-3-Hexenyl butanoate	0.005	449.28	—	—	149.28	—	146.69
(Z)-3-Hexenyl hexanoate	0.007	—	—	—	105.83	—	55.02
4-Hexen-1-yl hexanoate	0.012	275.53	—	61.29	—	—	—
Butyl acetate	0.066	—	120.27	17.93	—	—	—
Butyl butyrate	0.01	274.22	17.47	132.12	35.01	—	49.29
Ethyl (Z)-7-octenoate	0.003	208.23	—	—	—	—	100.5
Ethyl acetate	5	3.16	4.33	1.12	0.77	0.46	1.01
Ethyl octanoate	0.005	451.69	—	183.24	46.75	—	26.71
Hexyl hexanoate	0.02	127.97	—	50.49	21.6	—	10.12
Octyl acetate	0.012	—	95.9	25.94	—	—	—
γ-Decalactone	0.011	—	691.01	541.2	—	—	—
δ-Decalactone	0.05	—	13.96	10.63	—	—	—
Cedrol	120	—	—	0.01	—	—	—
Linalool	0.006	—	99.34	132.71	—	—	—
β-Dihydroionone	0.004	—	—	—	—	50.13	18.99

Note: Relative odor activity values were calculated using odor detection thresholds from the published literature, not measured directly in Younai. The odor threshold data were cited from Refs. [13,14,15,16].

## Data Availability

The data presented in this study are available on request from the corresponding author due to the fact that the data contain unprocessed original detection data and related experimental parameters.

## Supplementary Materials

The following supporting information can be downloaded at https://www.mdpi.com/article/10.3390/foods15101787/s1, Table S1: Sampling sites and basic fruit traits of test samples; Table S2: The 293 metabolites in Younai fruits; Table S3: The correlation coefficients of core flavor compounds in Younai fruits; Table S4: The *p* values of the correlation analysis of core flavor compounds in Younai fruits. Figure S1: Principal component analysis (PCA) score plot of volatile compounds across different sample groups. SB-GT: Shiban Wannai from Gutian County; CP-YX: Cuiping Younai from Youxi County; CP-GT: Cuiping Younai from Gutian County; WHJ-JO: Wanhuangjin from Jian’ou City; WHJ-LC: Wanhuangjin from Liancheng County. Other figure annotations are the same as above, Figure S2: Principal component analysis (PCA) score plot of metabolomic profiles across different sample groups and quality control (QC) samples.

## Abbreviations

The following abbreviations are used in this manuscript:

rOAV	Relative Odor Activity Value
HPLC	High Performance Liquid Chromatography
HS-SPME-GC-MS	HeadSpace Solid Phase Microextraction–Gas Chromatography Mass Spectrometry
UPLC-QTOF-MS	Ultra Performance Liquid Chromatography Quadrupole Time of Flight Mass Spectrometry
SB	Shiban Wannai
CP	Cuiping Younai
WHJ	Wanhuangjin
JY	Jianyang District
GT	Gutian County
YX	Youxi County
JO	Jian’ou District
LC	Liancheng County
DAFB	Day After Full Bloom
TSS	Total Soluble Solids
TA	Titratable Acid
GC-O	Gas Chromatography–Olfactometry
