# Supplementary material for: Systematic Characterization of Flavor Profiles and Screening of Potential Key Aroma-Active Components in Prunus salicina var. cordata cv. ‘Younai’
Source: Foods. 2026 May 18;15(10):1787. doi: 10.3390/foods15101787 (PMC13205203; doi:10.3390/foods15101787)
Supplement: Supplementary file 1 [file foods-15-01787-s001.zip › foods-4264699-supplementary.pdf]

Systematic Characterization of Flavor Profiles and Screening of Potential Key Aroma-Active Components in *Prunus salicina* var. *cordata* cv. 'Younai'

Lijuan Fu [1,2,†], Wenjing Liu [1,†], Lihua Ren [1], Xiangxin Lin [1], Jia Guo [1], Hao Chen [3], Faxing Chen [2,*] and Sun'an Yan [1,*]

[1] Institute of Quality Standards & Testing Technology for Agro-Products, Fujian Academy of Agricultural Sciences/Fujian Key Laboratory of Agro-Products Quality and Safety, Fuzhou 350003, China; fulijuan_f@163.com (L.F.); liuwj163163@163.com (W.L.); rlhlyx123@163.com (L.R.); linxiangxin2008@163.com (X.L.); 15960102868@163.com (J.G.)
[2] College of Horticulture, Fujian Agriculture and Forestry University, Fuzhou 350002, China
[3] Fujian Green Food Development Center, Fuzhou 350003, China; chenhao007321@sina.com
* Correspondence: cfaxing@126.com (F.C.); yansunan1982@163.com (S.Y.)
† These authors contributed equally to this research.

Supplementary Figures

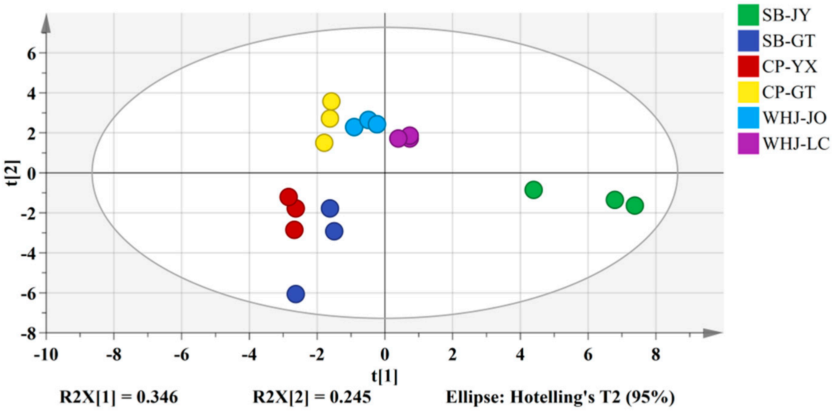

Supplementary Figure S1. Principal component analysis (PCA) score plot of volatile compounds across different sample groups. SB-GT: Shiban Wannai from Gutian County; CP-YX: Cuiping Younai from Youxi County; CP-GT: Cuiping Younai from Gutian County; WHJ-JO: Wanhuangjin from Jian'ou City; WHJ-LC: Wanhuangjin from Liancheng County. Other figure annotations are the same as above.

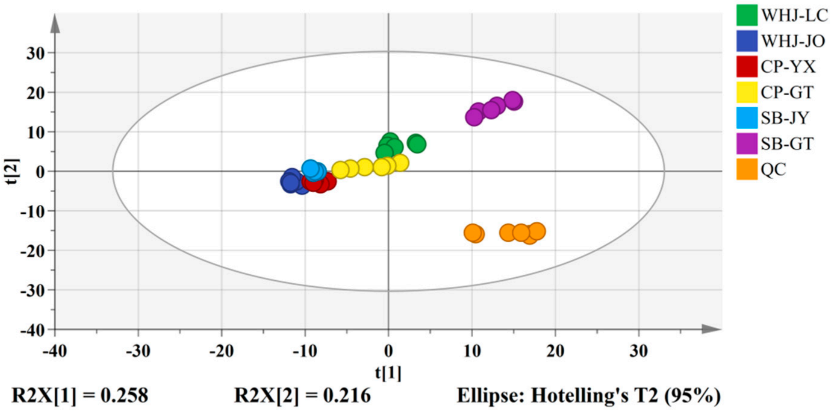

Supplementary Figure S2. Principal component analysis (PCA) score plot of metabolomic profiles across different sample groups and quality control (QC) samples.

Supplementary Tables

Supplementary Table S1. Sampling sites and basic fruit traits of test samples.

Sample Code	Cultivar Name	Sampling Regions	Production Entity	Fruit Longitudinal Diameter (mm)	Fruit Transverse diameter (mm)	Single Fruit Weight (g)	Geographic Coordinates	Sampling Time
SB-JY	Shiban Wannai	Jianyang District, Nanping, Fujian	Jianyang Germplasm Repository	55.75±2.27	64.06±1.85	102.01±3.04	27.35°N 118.02°E	2024.08.15
SB-GT	Shiban Wannai	Gutian County, Ningde, Fujian	Jiusongshan Family Farm	55.48±2.46	64.36±2.09	96.40±4.21	26.63°N 118.71°E	2024.08.14
CP-YX	Cuiping Wannai	Youxi County, Sanming, Fujian	Cai Heli Family Farm	62.01±2.07	65.39±2.18	124.22±3.66	26.29°N 117.72°E	2024.07.29
CP-GT	Cuiping Wannai	Gutian County, Ningde, Fujian	Zheng Zhonghai Family Farm	61.00±2.97	66.00±1.08	118.52±4.57	26.68°N 118.77°E	2024.07.30
WHJ-LC	Wanhuangjin	Liancheng County, Longyan, Fujian	Yinglian Family Farm	55.64±1.91	64.37±1.15	100.40±3.22	25.88°N 116.72°E	2024.08.30
WHJ-JO	Wanhuangjin	Jian'ou District, Nanping, Fujian	Liao Chunsheng Family Farm	55.61±2.57	63.52±2.58	99.92±2.91	26.82°N 118.33°E	2024.08.31

Supplementary Table S2. 293 metabolites.

Peak name	Retention time (min)	Mass charge ratio （Da）	Qualitative results	SB-JY			SB-GT			CP-YX			CP-GT			WHJ-JO			WHJ-LC		
				SB-1	SB-2	SB-3	SB-4	SB-5	SB-6	CP-1	CP-2	CP-3	CP-4	CP-5	CP-6	WHJ-1	WHJ-2	WHJ-3	WHJ-4	WHJ-5	WHJ-6
M181T5	0.0882	181.0708	Dulcitol	21641	180414	88378.3	55584.2	68565.8	23863.7	8298.15	90558.9	56278.8	27962.5	61923.6	12619.8	24549.7	42086.2	26981.1	19703.9	108268	201033
M389T31	0.5168	388.9366	5-phosphoribosyl-1-diphosphate	2777.53	3745.55	2968.29	2808.26	2402.56	2838.1	2464.64	1992.21	2764.9	3219.96	3043.46	1948.06	2217.5	2207.13	2097.67	2252.21	2244.91	2084.6
M154T36	0.6008	154.0618	Histidine	45.1984	55.3809	105.278	127.7	82.5479	97.3829	67.1723	163.837	154.583	71.4013	90.8986	149.801	88.0858	278.263	177.696	40.9008	35.7608	41.202
M113T38	0.6298	113.0346	3-Cyano-L-alanine	67418	46063.3	65499.5	74713.7	95463	70821.7	67191.9	66127.9	78781.2	65362.1	65106.8	73431.8	46282	66564.3	63910.8	34186.8	36453.8	36085.1
M132T38	0.6301	132.0297	Dl-Aspartic acid	5716.57	8838.77	6557.45	5929.61	6106.98	5039.46	14693	15124.5	11707.6	8007.39	9831.53	10228.6	8762.68	11600	9213.06	6626.18	5798.93	4411.03
M131T38	0.6307	131.045	L-Asparagine	153737	138396	186586	145343	191487	146632	148972	120375	156575	148794	148007	169841	76734.3	97546	120689	95908.1	110766	89846.3
M259T38	0.6364	259.0217	Fructose 6-phosphate	8782.6	8657.89	8032.18	12154.2	9806.51	11737.7	3412.54	3755.65	2974.53	6108.65	6076.68	5208.2	3263.01	4049.78	3433.83	11559.1	10035.4	5273.67
M261T38	0.6377	261.0365	Sorbitol-6-phosphate	1565.12	1580.47	1803.54	1481.27	1529.81	1171.89	1256.54	1694.61	939.409	857.612	633.593	740.045	2145.01	2319.36	2213.37	3097.84	2716.01	1770.02
M104T39	0.6447	104.1063	Choline	13853.5	13155.7	9019.22	38520.6	57170.6	42974.8	6231.26	14642.9	14221.3	29918.5	40307	30352.4	14193.2	13923.7	11097.4	17942	24425.7	22377.3
M145T39	0.6518	145.0614	Glutamine	3514.16	4235.66	4386.45	2300.64	1639	2243.58	3745.93	3449	3742.87	3093.62	3363.21	3689.27	4617.51	2919.06	3179.61	4078.35	2969.12	3438.6
M421T39	0.653	421.0738	Trehalose 6-phosphate	2315.39	2213.54	2742.6	2985.57	2815.3	3161.41	951.987	1157.89	1530.24	1317.68	2699.1	1942.23	570.112	604.272	631.532	2518.93	1985.02	2280.23
M132T39	0.6545	132.0653	trans-3-Hydroxy-L-proline	331.223	276.041	253.898	1913.11	2949.94	2132.46	126.137	213.086	206.361	408.956	437.212	399.056	215.494	292.767	200.917	540.085	742.415	612.685
M293T39	0.6556	293.0979	Tuliposide B	2451.87	1605.38	2506.03	3475.09	2465.6	2713.1	2899.09	2474.74	2881.35	5173.79	8512.18	6397.05	3908.05	2551.12	2851.7	734.245	1183.29	1074.26
M118T40	0.659	118.0506	Threonine	430.893	402.787	365.017	113.274	257.45	590.835	258.67	331.155	170.814	311.096	391.774	294.572	167.026	156.709	241.187	154.481	462.484	782.548
M209T40	0.6592	209.0297	Mucic acid	3098.7	3739	2281.46	2643.46	1721.47	3534.96	4629.25	3472.31	3440.24	1970.82	1973.28	2279.22	1260.82	1815.84	1821.06	2551.22	3058.81	1730.68
M195T40	0.6602	195.0497	Gulonic acid	111924	165001	131039	76159.5	103843	89069.5	140489	166001	184855	85576.1	109667	84593.2	118784	192527	137342	146779	175336	211437
M128T40	0.6632	128.0348	Pyroglutamic acid	6497.56	8469.35	8234.49	4807.37	4711.35	4528.64	6661.11	5789.57	5546.9	9131.79	9086.2	8399.88	6135.93	5908.93	5235.72	5302.59	4742.83	7313.07
M146T40	0.6653	146.0452	L-Glutamate	18969.2	14013.3	16982.2	13975.4	11603.2	15306.7	16394.4	16621	16250.2	21078.1	25942.6	22579.9	17082.9	13533.8	16345	21576.4	16359.7	17876.5
M182T40	0.6697	182.0744	Mannitol	17587.4	17413	18948.5	15176.6	18923.4	14424.2	14335.6	15916.4	11223	23034.4	19063.3	14295.8	17540.6	23216.1	19335.8	16255.5	20851.4	19906.7
M181T40	0.6742	181.0702	Harman	602023	846919	765971	729422	499993	764181	769153	610085	621830	723265	737208	689959	584508	487530	594439	709594	827383	837435
M369T41	0.6764	369.1201	sinapaldehyde glucoside	1355.59	1364.73	1965.47	2070.57	1783.95	3646.23	1893.88	1720.75	849.675	2127.56	1932.47	2211.94	1063.36	1609.11	1038.53	3214.71	2222.12	1446.61
M148T41	0.6777	148.0605	L-Glutamic acid	1540.04	1459.28	1264.95	6116.88	10791.4	7934.88	1094.36	1692.87	1617.74	5129.41	5703.91	3906.58	1698.37	1739.94	1584.88	4321.83	5584.06	4462.52
M165T41	0.6812	165.0398	Xylonic acid	35879.4	45193.7	47210.8	20596	22547.7	21902.6	38856	33833.2	41802.1	12893.1	9695.37	9610.62	24915.9	29159.8	26312.3	28130	30690.3	30998.1
M179T41	0.6826	179.0553	Gulose	11556.2	9746.17	12703	5780.96	8203.88	6409.05	7463.82	7795.04	7667.58	6709.65	7496.96	7830.78	6704.53	6435.99	7303.81	9027.53	10727	9663.78
M146T41	0.6862	146.0776	L-trans-5-Hydroxy-2-piperidinecarbo xylic acid	1191.61	1036.25	1249.07	5293.63	10854.6	7996.27	1036.27	1216.42	1255.06	2105.07	2126.02	2251.42	1170.81	1022.16	1247.08	6071.42	7539.44	6226.13
M399T41	0.689	399.1399	S-(5'-Adenosyl)-L-methionine cation	169.401	141.325	110.884	179.739	488.118	331.033	125.137	212.225	161.063	273.172	349.024	353.555	244.245	232.109	246.468	668.609	729.281	730.451
M165T42	0.6943	165.0751	D-(+)-Fucose	1483.39	1330.84	1335.37	20856	32522.7	27823.4	947.588	841.316	904.329	4640.25	4567.85	3398.18	1714.99	1793.43	1577.8	6925.18	7402.21	6068.91
M135T42	0.696	135.0283	L-threonic acid	92515.4	136797	140133	535.403	644.364	77852.7	910.529	73555.7	79154.6	114762	86387.3	84793.5	116.345	76567.9	159145	120978	138256	131431
M118T43	0.722	118.0856	Valine	1446.79	1346.16	1033.25	5112.67	6623.11	4441.19	1476.7	1938.53	2051.01	3149.9	2958.41	3151.27	1645.78	1704.48	1723.66	2841.88	3100.07	3132.73
M116T44	0.7286	116.0681	L-Proline	16661.5	15202.6	9043.45	165246	210829	165517	9430.39	24394.6	24448.1	80671	67251.1	62092.7	21787.8	20502.1	22739.3	25441.5	32030.1	34522.2
M341T45	0.7571	341.1073	Trehalose dihydrate	227921	177696	213187	354728	299607	258032	397550	412991	532823	334619	394964	296862	178962	163272	231186	329538	309072	377181
M271T46	0.7611	271.0813	Alloimperatorin	998.494	920.371	740.222	2532.33	6316.28	5187.59	745.73	1092.76	1120.58	3472.22	3060.66	2590.21	791.225	890.955	883.258	2708.19	3324.86	2991.06
M527T48	0.7943	527.1512	Glycan 3'-Galactosyllactose	638.704	643.913	700.284	1849.51	1421.31	1521.09	844.693	505.3	318.174	550.42	600.077	553.163	430.484	453.449	389.205	1141.14	1327.15	274.151
M173T48	0.7962	173.0088	Cis-Aconitic acid	12889.7	9097.75	11119.3	4391.96	6582.29	4995.75	6530.47	7816.32	6672.11	8469.48	8205.75	8283.62	8833.28	6613.56	9039.69	543.225	3370.62	3662.64
M115T49	0.8176	115.0028	But-2-enedioic acid	299840	282397	309039	282252	339360	248597	220204	226385	229445	345779	342625	329748	197247	217193	221784	246002	303506	322214
M325T50	0.8319	325.1128	D-Turanose	7390.24	6802.75	5218.77	65615.5	112426	87393.5	11521.5	15054.2	14614.3	77212.1	59678.4	52887	11176.3	11498.6	10929	33355.9	30531.8	27914.6
M365T54	0.8982	365.1053	α-1,4-Galactobiose	14246.5	15811.3	13210.2	33991.4	64332.9	65952.5	8104.59	2670.22	2493.78	8469.2	8644.91	6575.92	3269.62	2632.2	3099.83	69753.8	60074.4	56912.7
M133T55	0.9171	133.013	Malic acid	211110	282396	263960	413043	312369	141923	378241	332083	345638	26517.7	308211	390491	26407.6	298177	237993	49685.9	407603	494520
M147T62	1.0369	147.0648	Glutamine	136	129.369	126.238	650.538	850.935	663.501	185.789	161.878	171.502	504.943	427.473	450.27	253.233	224.458	242.582	214.05	252.482	361.882
M181T63	1.049	181.0711	D-Sorbitol	21253	24540.6	25046.7	13035.3	16705.9	15610.4	18730.7	19391.4	23599.1	31001.3	29613	21656.5	17434	18916.7	17115.2	13186.2	25866.8	21481.7
M118T63	1.0541	118.086	L-Valine	618.983	604.062	721.644	2307.29	3653.28	3842.36	781.561	729.432	734.122	2159.43	1500.54	1777.91	611.067	623.614	763.28	1340.62	1317.89	1232.22
M341T65	1.0803	341.1074	Laminaribiose	31205.8	39840.3	33648	45410	42950.9	33264.6	82217	107770	118257	44119.1	53228.4	38617	22488.1	24497	27359.2	46752.1	68869.9	56968.2
M342T65	1.0811	342.1116	Cellobiose	1409.06	1666.33	1684.25	2295.37	1955.56	1590.96	3457.12	4171.44	4584.98	1902.18	2015.07	1719.91	1096.19	1084.55	1105.05	2595.11	2485.32	2152.61
M323T66	1.094	323.0281	Pseudouridine 5'-phosphate	223.92	175.03	297.459	221.794	201.635	117.73	60.0545	89.6885	97.889	288.375	198.183	282.455	120.402	124.372	126.646	39.6698	47.7859	44.3214
M179T66	1.0965	179.0555	Galactose	2638.29	2956.45	3457.93	3302.01	2408.5	2348.16	2453.85	2528.64	2776.39	2745.07	2732.71	2614.25	1991.12	2512.08	3076.48	2002.81	2025.62	1740.99
M130T67	1.1181	130.0858	Cycloleucine	1309.55	921.198	1062.6	17439.6	30646.3	18616.2	1578.06	1367.92	1365.98	2639.09	1590.66	1936.08	691.696	739.962	707.706	1036.06	2294.56	1931.69
M527T68	1.1256	527.1584	Galα1-6Glcα1-2βFruf	1543.73	1249.35	2083.13	2880.41	4087.99	4644.1	972.826	393.307	367.519	979.029	760.386	618.793	425.362	400.153	487.795	3916.98	9536.76	4069.39
M289T68	1.1317	289.0914	2-Methyl-3-[(2S,3R,4S,5S,6R)-3,4,5-t rihydroxy-6-(hydroxymethyl)oxan-2-yl]oxypyran-4-one	166.852	148.704	644.146	622.136	552.582	457.821	645.837	422.356	355.743	1487.17	1073.1	1104.9	383.582	435.47	447.621	235.281	464.497	500.27
M522T68	1.1343	522.2027	Melezitose	122.155	154.648	244.159	741.242	3105.64	1742.28	455.391	307.375	313.285	3200.91	1766.71	1721.34	350.469	431.164	376.618	262.687	480.154	381.841
M545T68	1.1371	545.1323	5-hydroxy-3-(5-hydroxy-2,4-dimetho xyphenyl)-6-methoxy-7-[3,4,5-trihydr oxy-6-(hydroxymethyl)oxan-2-yl]oxy chromen-4-one	119.68	123.84	206.745	233.06	264.947	167.359	166.822	107.155	120.27	112.14	128.053	147.5	131.643	100.617	140.537	116.849	165.577	117.031
M1007T68	1.1399	1007.3273	Raffinose	6544.41	7637.33	7636.45	3700.3	5362.33	2532.42	44989.5	29687.7	22452.2	32181.2	18000.8	24999.1	10633.2	3473.66	9746.96	4362.31	4028.07	6904.81
M343T69	1.1541	343.1235	β-Lactose	199.492	133.966	341.217	157.041	1339.72	951.723	812.498	462.848	397.874	2777.97	1780.97	1562.61	415.438	463.757	453.6	332.85	537.312	664.879
M325T70	1.1596	325.1128	β-Gentiobiose	638.228	590.296	1180.98	4061.92	3895.66	2224.53	3237.47	1956.94	1651.74	13452.3	8858.58	8308.36	1948.63	2274.19	2337.16	1070.64	1943.58	1571.19

Peak name	Retention time (min)	Mass charge ratio （Da）	Qualitative results	SB-JY			SB-GT			CP-YX			CP-GT			WHJ-JO			WHJ-LC		
				SB-1	SB-2	SB-3	SB-4	SB-5	SB-6	CP-1	CP-2	CP-3	CP-4	CP-5	CP-6	WHJ-1	WHJ-2	WHJ-3	WHJ-4	WHJ-5	WHJ-6
M111T73	1.2169	111.0078	2-Furoic acid	83613.2	94534.4	104236	58080.4	84222.1	52315	101816	89038.9	91337.2	105043	86160.2	83741.9	92750.2	73678.6	80040.6	39999.2	64128.9	72778.4
M173T73	1.2178	173.0086	Aconitate	3935.21	5229.62	4567.38	4077.26	4983.48	2845.8	3738.13	4265.33	3873.3	4124.61	3905.78	4182.77	4301.91	4056.69	3847.29	2879.39	3819.26	2524.35
M133T78	1.2919	133.0138	Malate	2029.09	2913.84	2306.97	1845.61	1911.07	2283.41	1649.78	1438.48	1567.66	2596.56	2884.81	3049.48	1050.86	1162.07	1087.92	2094.14	2170.72	3168.39
M341T78	1.307	341.1078	isomaltulose	3536.15	5076.23	3703.17	5053.35	6331.36	5037.53	12108.2	14301.7	14452.1	6738.14	7687.83	6593.22	2809.52	3842.22	3357.75	8136.74	8615.13	9883.08
M503T82	1.364	503.1605	Levan	13786.5	16492.2	11895.4	17136.4	14158.2	19823.9	14646.8	15974.7	16756.7	11411.4	1162.25	5332.88	457.26	567	449.3	5022.41	1267.92	1164.98
M191T89	1.4859	191.0183	Citric acid	358543	379917	372158	406204	756554	428483	317212	407538	307354	326461	47804.4	522768	21682.9	107433	230584	377506	334476	286206
M111T89	1.4897	111.0077	Uracil	178683	145691	148529	153952	155904	125137	297342	189681	159702	25552	16637	335815	228998	178003	130533	108002	126075	124582
M191T113	1.8882	191.055	Quinic acid	1907.98	3264.38	3031.18	2003.84	2992.47	2430.34	1715.15	2231.92	1649.75	2603.34	2424.41	2333	1754.5	1147.54	1869.7	1661.92	2087.65	1901.14
M117T115	1.9088	117.0188	Methylmalonic acid	6991.1	8011.56	5482.62	5945.85	5404.15	5247.64	5664.68	7824.58	1777.36	3936.46	3026.07	3501.34	4483.57	8754.74	2992.98	1330.38	6293.49	3743.8
M341T118	1.973	341.1075	Trehalose	5935.05	6912.14	12905.2	9439.12	10313	5522.4	18774.2	20387.9	31739.9	12833.9	72081	8532.71	5099.69	5759.75	6228.82	9918.74	16497.7	30133.2
M711T118	1.9746	711.2193	Tetrasaccharides (Hex-Hex-Hex-Hex)	277.677	284.961	250.219	72.2308	89.2914	30.9315	235.851	76.2777	326.314	898.052	25.3799	116.088	391.707	250.575	189.352	117.06	296.633	144.462
M542T120	1.992	542.069	Cyclic adenosine diphosphate ribose	70.136	75.2382	62.5773	63.4725	123.989	100.765	63.4906	61.3572	125.783	62.6236	84.058	82.0471	87.4764	87.4405	84.7535	68.3379	72.7541	88.88
M664T120	1.9971	664.1165	α-Nicotinamide adenine dinucleotide	77.3776	87.2047	63.3389	64.2819	122.464	105.145	63.2647	252.169	177.86	178.812	195.64	198.74	111.595	128.972	106.499	75.2459	84.7634	95.3491
M229T121	2.0153	229.1544	Pro-Leu	2299.4	1440.01	467.102	1887.46	8462.24	4222.63	1323.45	3168.38	2779.85	9024.41	5568.2	7290.52	2618.69	3639.77	3048.16	2790.18	4632.98	3444.24
M182T121	2.0165	182.0792	L-Tyrosine	204.744	171.766	92.6387	538.939	1364.17	636.64	78.7121	621.33	635.696	563.385	644.76	758.189	501.96	936.16	555.426	210.548	338.58	488.741
M132T121	2.0185	132.1016	Norleucine	149.85	119.494	141.176	97.4499	2218.57	1068.88	97.6433	390.38	330.066	435.205	1022	1144.27	281.164	375.031	306.34	294.868	400.26	392.513
M243T122	2.0342	243.0614	Uridine	3145.66	2147.58	2387.78	977.312	2208.78	794.865	250.751	346.581	251.7	1429.12	2673.31	1851.37	1353.48	609.504	991.399	1510.1	646.836	1232.86
M180T122	2.0381	180.0659	Tyrosine	320.017	283.876	190.378	530.021	641.674	492.025	1530.11	2027.27	2674.84	708.64	1137.58	1046.41	3620.29	2208.96	3403.61	576.862	719.33	585.723
M481T122	2.0399	481.1162	Quercetagitrin	97.6719	89.7593	71.5995	72.5969	123.214	116.599	71.288	69.7965	70.3164	448.543	351.96	410.136	89.2791	82.5271	86.9527	103.969	164.884	101.912
M121T125	2.0907	121.0634	4-Vinylphenol	1610.98	1042	199.641	340.704	36063.4	20110.5	249.241	3015.6	2319.26	8031.64	8105.77	8044.67	2421.23	1698.04	1861.01	2526.56	4400.01	5154.31
M241T125	2.0907	241.1544	N,N-dimethyl-proline-proline betaine	3569.73	2001.16	95.4427	183.836	13481.2	6841.74	96.1814	4943.9	5220.87	18870.5	15193.8	15296.5	6769.94	7887.19	6585.23	4330.42	7090.26	6753.38
M147T130	2.1637	147.0293	L-alpha-Hydroxyglutaric acid	17187.1	15915.1	18921.7	15897.3	12167	10237	6794.11	4563.05	5972.63	25276.5	28147.7	29434.3	23494.6	27122.5	21788	12906.4	10375.6	9181.77
M268T130	2.1732	268.1028	Adenosine	3279.27	1336.53	83.6257	135.226	46847.2	27126.8	81.6063	739.069	404.663	31512.7	21666.4	25384.2	1048.26	1379.68	1117.27	850.447	1084.16	915.143
M282T134	2.2353	282.0834	Guanosine	2773.89	2526.01	2672.09	811.727	1597.06	925.419	126.963	238.561	122.291	1399.97	1565.09	1432.68	762.509	864.363	1061.66	162.403	147.411	265.935
M138T141	2.3559	138.0192	6-Hydroxynicotinate	721.08	851.522	903.205	309.69	829.845	393.864	801.779	727.716	766.535	849.016	742.818	802.562	1267.05	860.809	1059.4	376.731	538.778	646.378
M239T142	2.3697	239.0821	Cystine	73.8749	77.3367	88.071	62.5846	67.5314	39.8862	86.745	55.7566	88.1522	143.255	101.175	134.814	44.2834	11.1657	23.2176	22.7118	23.8358	45.3427
M161T147	2.4545	161.0787	L-Ala-Dl-Ala	202.041	192.909	238.434	902.463	1306.49	833.689	172.894	168.379	149.703	464.659	398.056	321.843	121.396	154.412	143.103	260.59	378.193	278.935
M153T160	2.6634	153.0188	2,3-Dihydroxybenzoic Acid	625.998	616.883	777.703	524.421	784.562	640.555	836.625	848.019	1023.57	338.15	499.393	861.61	1049.99	1155.61	1089.9	535.763	543.383	1088.17
M179T160	2.6672	179.0555	Sorbose	871.989	1096.49	841.127	988.01	1108.75	900.554	966.758	930.421	978.96	1037.02	1049.56	815.877	942.972	929.6	705.047	704.703	857.531	580.404
M315T160	2.6705	315.0716	Protocatechuic acid 4-glucoside	268.252	377.662	436.754	312.247	411.094	256.778	682.464	583.569	570.308	183.968	256.54	237.079	237.115	379.124	340.981	399.802	667.159	484.105
M230T163	2.7151	230.0809	5-(L-alanin-3-yl)-2-hydroxy-cis,cis-muconate 6-semialdehyde	81.8985	80.4668	75.5311	88.3602	111.012	82.4513	74.9767	83.782	75.7175	74.7804	85.8436	94.805	75.3401	73.9246	76.7548	82.2766	88.9953	82.7704
M443T164	2.7297	443.1659	8-(β-D-GlcO-)-3-methoxy-1-methyl-TH-pyrano[3,4-c]pyran-5-carboxylic acid methyl ester (1R,4aS,8S,8aS)	106.163	101.971	143.813	78.4744	304.688	210.777	74.5803	213.598	128.125	427.812	229.649	149.066	120.819	149.013	140.434	184.173	227.266	243.457
M137T164	2.7311	137.0237	2-Aminonicotinic acid	2341.25	3438.16	2651.9	2388.91	2413.26	1953.56	2506.94	3284.13	3217.68	2686.1	2459.36	2565.84	2026.19	2305.72	2682.56	1871.13	2120.49	2400.7
M299T164	2.7313	299.0761	4-Hydroxybenzoic acid glucoside	1256.4	1042.66	1139.23	1491.06	2282.91	2321.31	1200.77	1416.15	1967.61	1545.16	1113.07	1450.03	728.783	942.818	970.966	2117.12	3222.94	1510.7
M164T166	2.7611	164.071	Phenylalanine	3092.45	4211.73	4984.54	4480.6	6709.51	3713.88	12568.1	12911.4	9931.69	8205.87	10424	16309.4	4182.09	4222.29	3379.1	1504.87	2395.82	2345.39
M122T176	2.9275	122.0244	Picolinic acid	284.71	400.295	422.167	675.562	453.159	673.476	1153.54	196.702	516.795	522.995	220.181	721.687	382.203	700.63	1691.76	1269.74	494.115	462.109
M179T177	2.9463	179.0557	Glucose	573.788	737.239	771.117	384.306	832.901	411.586	557.202	632.135	465.53	369.994	299.801	413.168	652.113	315.883	396.572	441.66	745.258	744.621
M144T177	2.952	144.0452	4-Hydroxyquinoline	1125.5	1096.74	1809.28	687.682	1600.78	1431.87	1973.01	1614.71	770.764	614.605	692.703	716.115	3178.57	697.944	2768.48	1428.02	1246.51	1652.68
M315T181	3.0116	315.0699	Benzoic acid-2-O-hexosyl	3814.1	2542.07	2480.53	922.204	901.711	820.633	1993.49	2369.31	2205.17	2426.23	1891.8	3161.23	4268.68	4680.12	2643.36	1045.13	1391.47	1950.6
M134T182	3.033	134.06	Mandelonitrile	80.0692	103.525	68.3548	116.617	118.57	139.136	92.1922	159.474	66.1935	79.2281	91.069	111.064	75.1427	78.7523	72.1635	118.507	127.099	137.816
M421T182	3.0363	421.1675	Rhaponticin	403.806	403.067	392.558	379.449	1308.69	630.505	218.895	193.568	207.358	442.5	345.291	421.036	368.106	300.899	230.552	366.533	603.423	758.053
M529T185	3.0915	529.1809	Maltotriitol	64.2781	63.8677	63.7211	58.8016	61.104	64.2087	63.1059	64.0237	63.5697	68.9338	66.3319	64.2276	64.3926	59.0332	64.3771	64.2653	64.2242	65.0083
M167T190	3.1628	167.0341	Vanillic acid	6402.23	7325.16	7464.15	6822.29	9681.1	6311.84	15805.4	14654.4	13789.2	7512.52	6689.23	8354.49	6645.07	7734.95	8247.28	4240.41	6140.61	5944.87
M329T190	3.163	329.0865	Vanillic acid 1-O-glucopyranoside	6297.22	9183.41	10206.5	4835.32	10060.2	4443.6	7653.47	8577.7	8524.46	6275.34	4990.64	6461.1	3123.25	4434.6	4683.43	3182.38	5297.99	7325.8
M153T199	3.3111	153.0547	2,5-Dihydroxy benzoic acid	1661.72	2205.85	2833.38	2028.63	3027.3	1881.49	4210.81	3937.56	3515.15	2499.74	2295.43	3162.08	2052.95	2604.42	3545.04	1113.43	2541.56	2039.71
M315T199	3.3141	315.1069	Vanilloloside	3867.93	3811.24	5566.8	3054.92	7565.7	2831.54	8697.98	6816.94	6679.16	5083.02	5290.04	6371.23	2082.37	2614.56	4114.03	3302.34	3352.68	2912.46
M107T199	3.3219	107.0507	3-Hydroxybenzyl alcohol	305.705	323.274	324.887	324.565	701.852	611.629	141.058	187.615	101.573	198.929	160.915	198.202	227.485	249.871	245.549	308.729	437.091	305.514
M146T202	3.3661	146.0596	2-Hydroxyquinoline	81.8794	81.6948	63.0509	155.084	190.785	129.216	85.1694	113.467	92.2653	54.5924	96.42	116.383	180.095	261.447	210.001	101.002	135.531	108.326
M347T203	3.3862	347.0981	NCGC00380682-01	606.204	273.075	260.811	553.857	193.978	308.009	359.448	438.112	292.511	422.894	214.95	66.0616	165.411	122.37	118.708	335.38	203.338	203.166
M491T208	3.463	491.1375	Kaempferol-O-acetylhexoside	159.08	137.224	163.572	40.4402	364.795	243.577	56.5673	45.2774	46.9394	41.0829	73.4819	70.5168	74.639	65.0098	78.8791	229.64	368.637	341.032
M195T212	3.5294	195.0656	Salicyluric acid	563.94	683.16	437.424	359.43	514.886	369.846	723.183	782.006	588.042	617.709	733.484	905.814	376.775	472.261	301.922	301.463	295.874	355.813
M179T218	3.6414	179.0342	Nicotinuric acid	4310.08	4125.77	2735.94	5182.84	4502.28	3659.66	8430.07	4786.9	4906.73	5325.04	5803.51	6382.01	3738.14	3112.85	3050.3	3881.36	5477.15	5093.81
M354T219	3.6439	354.0899	1-Caffeoylquinic acid	19355.9	25488.7	34473.2	19747.4	26942.5	14448.2	23844.2	27423.8	28545	33729.3	28233.8	28389.5	12957.1	12778.4	11792.2	10784.7	21582.4	36526.6
M231T219	3.6447	231.0176	Fraxetin	773.639	612.912	730.808	5964.74	7595.5	5662.07	221.054	203.761	223.525	491.079	494.747	395.577	165.873	148.715	152.1	2522.09	3698.01	3410.35
M353T219	3.6475	353.0859	5-O-Caffeoylquinic acid	340108	382036	411017	392321	415809	275741	442573	468293	461855	501305	531592	552706	252757	249130	210342	198126	322330	287756
M707T219	3.648	707.1814	Chlorogenic Acid	1371.59	683.918	752.8	747.108	888.05	1203.29	1050.78	1167.99	937.329	3138.33	1088.61	2711.2	555.66	304.196	282.396	345.198	478.851	448.065
M146T223	3.7191	146.0582	Indole-3-carboxaldehyde	1061.64	878.713	1035.13	2963.93	5705.7	3367.39	4886.22	3926.6	3656.14	6837.84	5701.09	6076.17	3501.13	3772.86	3298.31	1358.1	2306.92	2372.08
M188T224	3.7259	188.07	L-Indole-3-lactic acid	1294.39	993.643	1363.48	30781.7	47416.9	25854.3	6712.57	5884.44	4774.1	14286.4	11181.6	11551.3	5561.08	6110.27	5197.19	2463.57	4157.14	4265.54
M203T224	3.7286	203.0817	Tryptophan	3420.31	2969.73	3341.54	4803.34	5932.6	6736.14	10695.9	12381.3	10771.6	3311.54	4665.82	3300.61	8032.4	17482	11604.6	2002.14	3106.79	1454.19

Peak name	Retention time (min)	Mass charge ratio （Da）	Qualitative results	SB-JY			SB-GT			CP-YX			CP-GT			WHJ-JO			WHJ-LC		
				SB-1	SB-2	SB-3	SB-4	SB-5	SB-6	CP-1	CP-2	CP-3	CP-4	CP-5	CP-6	WHJ-1	WHJ-2	WHJ-3	WHJ-4	WHJ-5	WHJ-6
M307T226	3.7745	307.0848	(-)-Gallocatechin (GC)	110.181	111.076	101.022	139.447	287.33	226.921	145.426	147.056	89.7433	180.674	147.093	194.466	65.505	112.843	81.9333	140.081	137.47	164.79
M491T228	3.7948	491.1285	Cyanidin-3-O-(6"-acetylglucoside)	92.2485	127.434	118.639	167.055	192.259	138.26	75.8355	93.9798	89.0161	77.5108	83.9667	79.9991	83.1068	66.2906	82.1625	148.5	210.039	216.05
M315T229	3.8203	315.0977	Cajanol	220.083	300.098	195.766	190.059	387.752	129.737	323.923	322.167	322.54	64.5716	69.359	90.162	289.632	42.1021	190.134	105.118	329.609	245.079
M163T233	3.8847	163.0393	Coumarinic acid	1032.56	813.02	1450.38	517.741	1014.84	799.929	420.617	336.098	245.706	703.603	609.771	796.057	1530.1	1351.44	1444.11	929.777	1231.33	1018.04
M341T238	3.9699	341.0864	1-O-Caffeoylglucose	262.455	322.098	328.277	307.217	436.842	330.219	467.032	685.961	565.091	543.15	546.429	639.88	452.055	277.434	261.196	334.881	441.368	553.56
M163T241	4.0135	163.0388	D-4-Hydroxy-2-oxoglutarate	116.069	113.647	114.043	833.553	704.85	624.249	137.556	116.987	102.351	274.231	343.895	380.302	141.086	130.298	143.257	317.726	333.931	330.641
M577T254	4.24	577.1326	Procyanidin B2	481761	464625	534832	426572	506066	483644	553206	561139	418446	606890	452987	541790	323496	358050	279878	154803	341774	410626
M254T255	4.2433	254.0355	Kinetin	119.601	123.848	184.546	273.125	146.149	275.534	73.9013	72.8644	76.9533	73.3091	82.7875	77.1358	77.4861	75.5079	76.5606	166.649	348.449	338.529
M165T266	4.4333	165.0551	4-Hydroxyphenyl-2-propionic acid	1542.99	1435.42	2092.27	1214.29	2293.28	1219.16	848.908	950.564	799.391	1180.28	1365.29	1407.1	2019.58	1976.58	2019.96	1283.95	2383.63	2797.02
M327T266	4.4336	327.1073	Dihydromelilotoside	5311.7	4385.22	4997.38	4432.79	4710.57	4936.5	2016.33	2068	1630.41	2845.58	3708.06	3421.86	4994.36	3973.84	4391.71	6494.6	6516.05	7418.79
M450T268	4.4734	450.1966	Benzyl 6-O-beta-D-glucopyranosyl-beta-D-glucopyranoside	67.9806	85.659	93.1216	51.0937	466.313	333.332	322.332	316.162	269.876	628.064	515.913	497.658	319.865	391.217	310.948	113.91	136.824	137.074
M209T273	4.5419	209.0808	α-Benzylsuccinic acid	105.7	104.872	98.735	268.237	571.691	335.392	283.325	219.594	215.934	517.154	455.864	537.402	211.452	258.907	228.9	145.326	246.062	246.354
M215T274	4.5607	215.1059	Harmaline	89.2117	77.3823	86.027	273.387	218.746	200.102	107.211	108.237	70.019	135.609	146.055	167.514	84.3493	98.4245	76.3	144.181	176.792	151.579
M487T277	4.6179	487.1436	alpha-D-Glucopyranoside	12769.6	7264.55	16862.2	3686.18	4270.51	2798.71	20397.1	22990.5	25113.2	6070.55	4814.56	4739.36	11123.5	8992.47	14707.5	5933.46	9450.51	6513.97
M337T279	4.6436	337.0915	3-p-Coumaroylquinic acid	5396.12	4365.2	6487.15	4003.61	4933.54	3839.59	4673.69	4900.47	4888.85	5480.98	4348.49	4625.79	3720.96	2758.17	3493	3490.35	7028.99	5425.96
M577T279	4.6436	577.1246	trans-Resveratrol	73313	53514.3	87836.8	125270	162683	142426	138721	151630	135152	172351	167580	170043	60503.1	44981.6	46348.3	34549.4	67959.6	56083.9
M163T279	4.6437	163.0393	4-Coumaric acid	1677.2	1230.76	1287.41	1898.89	1981.87	1526.95	1476.64	1629.98	1771.22	1744.42	1870.24	1984.4	1505.74	1101.73	1136.85	1137.84	1613.96	2107.56
M338T279	4.6464	338.0955	Coumaroylquinic acid	394.088	251.487	447.045	353.715	316.76	388.974	253.816	370.957	275.841	499.179	488.893	549.527	236.084	120.431	167.19	353.972	422.796	341.994
M358T279	4.6579	358.0807	3-O-Coumaroylquinic acid	67.6497	62.2319	66.1852	75.0562	103.555	70.84	68.0717	61.3672	68.376	65.9844	71.5837	64.8391	64.6611	68.5153	66.612	68.2439	92.9596	78.2361
M409T280	4.6594	409.0915	Moluccanin	231.905	179.121	226.756	121.506	330.107	335.216	366.413	383.549	292.307	689.715	636.835	670.581	215.358	225.555	200.623	215.529	267.986	265.013
M491T286	4.766	491.1372	Cimifugin 4'-O-beta-D-glucopyranoside	248.792	172.632	232.226	541.074	568.613	230.211	64.5242	65.965	59.5578	122.29	109.359	89.9	127.567	143.413	116.878	499.597	659.158	616.868
M343T287	4.7858	343.1022	NCGC00380817-01	569.438	438.12	451.379	431.235	512.954	376.839	719.974	881.065	568.952	545.87	407.409	506.889	396.479	319.411	525.989	316.229	466.461	393.448
M431T288	4.8028	431.1541	Apigetrin	1261.13	1069.49	1665.91	957.57	1128.61	715.231	11380.4	25259.9	15398.2	2303.56	1888.15	2204.59	2761.66	2589.22	3489.85	6382.24	10136.2	11594.8
M477T288	4.8034	477.1596	Phenylethanoid glycosides	684.631	548.201	646.258	335.78	362.67	403.379	3889.78	6570.41	4806.6	666.278	525.809	614.805	539.79	652.626	683.69	3305.32	5347.86	3751.15
M595T289	4.8098	595.1642	Pelargonin	566.615	555.731	582.963	1024.76	1431.69	841.465	991.731	1146.42	907.361	850.475	570.882	0	359.072	0	0	407.326	688.053	576.844
M341T290	4.8356	341.0869	Caffeic acid hexoside	741.599	803.984	750.586	654.045	650.926	630.78	1020.81	1086.98	852.016	956.287	721.356	856.703	1033.56	878.203	727.795	435.069	663.236	744.829
M465T293	4.8871	465.1008	Cyanidin 3-glucoside	23722.9	21460.3	24630.4	45509.7	79520	46221.4	70892.1	67308.2	61446.3	46633.8	37146.5	30541.4	11723.1	13214.8	10720.4	11556.6	25817.1	27530.2
M467T295	4.9119	467.1137	(2R,3R)-2-(3,4-dihydroxyphenyl)-5,7-dihydroxy-3-(β-D-glucopyranosyloxy)-2,3-dihydrochromen-4-one	455.233	422.229	477.875	3103.15	4885.08	2584.1	479.674	457.086	486.968	925.328	715.017	689.23	301.465	279.728	300.435	520.103	805.728	831.898
M289T300	5.0018	289.07	Epicatechin	865815	668126	963367	753391	1082890	728679	892360	899048	835441	919795	1040940	999545	581447	503566	491565	340120	534493	563673
M353T309	5.1567	353.0865	trans-5-O-Caffeoylquinic acid	2144.97	1797.37	2302.19	2634.69	3546.33	3085.62	2574.04	2358.49	2463.99	6895.87	7507.92	8291.63	2048.32	1273.96	1451.13	2244.87	3394.49	2668.13
M163T312	5.1951	163.0394	2-Hydroxycinnamic acid	3536.5	3152.13	4038.17	2035.6	2768.24	1839.17	2656.8	2251.19	2002.69	2112.43	1593.33	2184.38	3751.59	3599.31	3928.15	2954.08	3619.9	4120.21
M325T312	5.204	325.0916	Melilotoside	1002.25	1032.32	1277.54	712.402	803.591	649.628	780.547	616.797	804.092	710.845	828.024	666.686	1221.89	1809.45	762.096	957.274	1248.94	1298.27
M369T317	5.2906	369.0815	Fraxin	8021.31	2100.1	1574.88	1256.08	1700.84	689.092	1483.16	995.775	834.605	8645.48	7110.57	6350.79	1036.52	2624.03	2243.62	946.331	1840.13	1701.59
M177T320	5.3395	177.0533	Coniferaldehyde	1444.6	1270.32	1577.47	536.932	966.261	354.486	1040.84	760.263	834.147	1043.39	859.838	994.826	1029.93	1152.02	1119.16	731.337	1323.1	423.824
M865T321	5.349	865.1946	Procyanidin C1	82758	86348	146303	103245	140007	114817	105236	122722	142047	121164	148011	140161	46053.8	76012.7	65936.9	35149	99919.7	77764.3
M145T323	5.3823	145.0289	A-Ketoglutaric acid	42.6625	31.9635	15.5718	20.3251	21.1397	57.7075	38.8942	0	0	136.048	82.0744	17.105	25.339	51.4354	118.155	98.4513	116.719	64.6218
M399T327	5.4498	399.0925	Scopolin	405.345	238.762	281.951	79.0574	117.381	95.189	137.549	120.817	260.225	127.187	227.167	40.302	79.0736	26.6402	62.2031	214.564	86.0926	198.085
M376T327	5.4527	376.1593	3-[2-(β-D-Glucopyranosyloxy)-4-methoxyphenyl]propanoic acid	66.6655	59.197	61.9442	280.38	383.661	257.857	80.775	85.7212	62.0193	168.533	119.91	129.654	91.8854	91.7419	85.0846	72.7	73.204	92.5554
M354T332	5.5329	354.0899	5-Caffeoylquinic acid	398.838	466.323	522.572	218.754	495.185	300.744	536.517	345.72	563.083	477.744	512.39	533.422	406.063	453.535	162.526	236.108	418.452	503.955
M369T335	5.5817	369.1162	Feruloyl quinic acid	102.532	97.6378	92.1961	191.203	204.955	150.66	93.3711	90.4975	90.5126	101.186	105.574	108.884	95.4736	92.0729	92.1053	95.0137	139.821	119.819
M541T337	5.611	541.152	3,5-dihydroxy-2-(4-hydroxyphenyl)-8-isopentenyl-7-(β-D-glucopyranosyloxy)chroman-4-one	77.0519	78.3177	76.7228	74.1323	86.1921	90.3756	78.6459	84.9988	71.7706	70.9552	76.3175	82.1107	74.3151	75.6828	76.4338	74.25	82.1975	80.847
M367T337	5.6162	367.1019	(1R,3R,4S,5R)-1,3,4-trihydroxy-5-[(E)-3-(4-hydroxy-3-methoxyphenyl)prop-2-enoyl]oxycyclohexane-1-carboxylic acid	3143.28	2992.92	3970.59	4548.61	5728.96	4099.56	4118.69	3262.73	6513.75	7068.98	7107.63	7313.94	3135.13	3477.91	3549.85	2450.96	3464.71	4348.9
M475T342	5.7064	475.192	Amygdalin	170.993	149.094	131.441	1003.57	1310.39	665.35	547.145	781.153	756.878	2899.4	1907.43	1936.72	290.969	302.33	264.275	248.535	365.943	380.383
M261T342	5.7074	261.0288	5-Methylthioribose 1-phosphate	338.422	310.715	217.775	625.997	800.272	638.75	129.852	138.97	106.143	102.691	113.844	186.891	101.878	97.0309	96.6346	538.492	850.743	922.426
M577T351	5.8559	577.1326	Procyanidin B5	6323.19	6311.44	7681.21	8037.82	14080.9	8640.86	15149.8	8800.97	15369.2	16724.6	9255.32	8189.56	3152.62	3617.52	6080.78	3292.3	4907.64	5592.11
M447T356	5.9319	447.0921	Cynaroside	14.7582	743	728.832	367	407.531	694.254	14.4081	6548.07	18.1303	0	0	0	809.158	543.01	591.59	2844.02	3105.21	3451.6
M449T359	5.9888	449.1069	Kaempferol -3-glucoside	73.8388	81.6947	76.3885	76.2891	89.8718	73.5801	76.602	76.16	79.3145	72.8499	70.971	74.2729	73.3895	73.9009	73.7169	79.8914	79.8765	84.2625
M757T361	6.0189	757.2171	Kaempferol-type flavonol trisaccharide-1	30.1336	29.97	29.8544	30.2268	30.313	30.6865	29.6406	29.6599	30.8778	26.9368	28.2891	30.1248	30.1024	30.223	30.1647	29.3124	29.8662	30.9678
M461T367	6.1119	461.1284	Lactiflorin	14423.8	13898.3	13778.6	7720.23	4743.03	2946.72	3400.6	6677.31	4548.57	8881.28	7407.55	6618.48	6639.43	7188.31	4810.41	2734.25	2880.88	4291.25
M577T379	6.3179	577.1321	Procyanidin	153585	117416	148528	132403	177648	141743	180591	115202	162713	101073	78093.6	84343.7	83521.6	51083.3	78186.3	43259.2	59495.8	75811.1

Peak name	Retention time (min)	Mass charge ratio （Da）	Qualitative results	SB-JY			SB-GT			CP-YX			CP-GT			WHJ-JO			WHJ-LC		
				SB-1	SB-2	SB-3	SB-4	SB-5	SB-6	CP-1	CP-2	CP-3	CP-4	CP-5	CP-6	WHJ-1	WHJ-2	WHJ-3	WHJ-4	WHJ-5	WHJ-6
M420T386	6.4341	420.186	β-D-Glucopyranoside	505.927	496.582	576.929	13878.8	10579.9	6222	9335.78	11555.3	9865.55	16520.3	10679.2	11381	2735.7	3428.12	3007.43	6555.62	11002.9	10031
M597T387	6.4513	597.1632	Isoeriocitrin	105.144	88.7472	128.046	102.539	88.6635	90.4285	93.5255	80.6109	80.4065	84.68	190.494	147.218	100.963	103.969	108.21	79.9423	80.9123	83.9352
M355T394	6.5599	355.1016	trans-p-Feruloyl-beta-D-glucopyranoside	1050.66	1919.99	1984.63	898.253	1048.78	476.571	1314.14	987.659	1714.72	1525.28	1437.93	1543.9	2173.34	2199.75	2270.41	1221.14	1994.1	1703.96
M593T404	6.7329	593.1493	Kaempferol 3-rhamno-glucoside	44.7435	71.2749	29.3313	31.8106	49.2031	40	130.204	113.946	16.7527	18.1557	16	32.2103	0	0	0	52.2378	13.1618	53.5059
M449T415	6.9135	449.1069	Isoflavone glycoside	21575.4	19452.8	27901.9	26183.6	44367.5	24008.2	23777.4	26070.3	24001.5	19158.8	22327.3	23984	11075.8	9822.46	11286.9	5406.95	7069.03	8813.58
M447T416	6.9308	447.1487	Sakuranin	33831.1	29685.8	32604.1	18868.4	19395.5	13687.2	112670	95241	119353	34488.8	38059.7	39902.1	25420.6	22455	25512.6	53071	102976	105040
M341T421	7.0201	341.118	Sphalleroside A	1728.94	1557.62	1783.52	1169.43	2064.71	1234.72	1926.77	1610.75	1755.85	1726.32	1818.45	1886.94	2057.37	2051.84	1438.86	1201.2	1560.96	1693.22
M593T431	7.1753	593.1494	Kaempferol 3-neohesperidoside	24.9899	50.0469	37	36.4164	154.37	63	127.169	266.901	296.633	115.68	71.4304	38.2403	123.919	25.2982	78.5304	30.6381	94.4759	75.8137
M385T431	7.1908	385.1055	1-O-β-D-glucopyranosyl sinapate	229.972	94.303	195.117	31.9895	52.6964	34.7467	1304.21	1288.03	1319.82	110.686	103.933	99.3741	744.497	908.765	657.433	13.1699	105.479	87.5812
M329T446	7.4288	329.0869	Vanilloyl glucose	2202.47	1356.72	1765.57	1201.8	1431.55	984.221	1508.57	1419.34	1374.52	820.07	996.828	930.883	988.326	518.485	882.606	676.896	1062.83	1310.57
M245T452	7.5314	245.081	Torachrysone	5225.86	4677.28	4721.96	4333.85	5085.73	4609.47	5951.73	3474.79	3899.23	5235.08	5478.62	4462.59	2819.58	3603.73	3324.03	2089.06	2419.77	3615.74
M289T452	7.5332	289.0699	Leucopelargonidin	368809	193517	349945	277667	306899	245214	341696	349820	304582	370362	463228	350653	125694	191373	166592	116969	133489	138880
M579T454	7.5605	579.1495	Cianidanol	841.495	859.475	497.387	536.16	727.824	345.957	690.031	594.729	527.584	744.31	689.858	276.458	88.4769	41.2728	146.085	15.8952	230.5	209.856
M139T454	7.5608	139.0384	Gentisate aldehyde	5190.88	4168.03	4153.94	10145.1	14193.6	10045.5	6981.02	5855.3	6033.13	16563.3	14216.4	12092.1	3023.51	3402.88	2700.14	4824.11	6901.26	7225.52
M449T464	7.7272	449.1073	Pelargonidin 3-glucoside	3230.31	2930.59	3772.59	742.995	1149.63	1801.66	4166.91	3439.18	3152.44	2867.59	2801.58	2898.9	2107.36	2041.22	2070.58	999.561	674.394	1282.17
M517T493	8.2191	517.097	5-Carboxypyranocyanidin 3-O-β-glucopyranoside	45.5407	45.5023	45.5824	47.0143	46.6805	45.7644	46.1321	46.0147	46.2666	45.1769	46.0712	46.1019	45.5023	45.6502	45.7329	45.8944	45.3	45.3481
M421T525	8.7542	421.044	4-(Methylthio)butylglucosinolate	105.638	77.7153	84.5536	191.695	266.346	250.705	65.299	64.148	65.6956	63.5242	80.3251	99.1591	65.49	65.6827	66.5754	165.348	363.609	402.163
M285T543	9.0546	285.0627	Physcion	345.163	310.729	287.168	1146.87	1568.14	1120.91	192	144.675	128.267	61.2961	145.483	127.076	81.759	75.3743	78.5975	499.696	658.256	652.647
M521T553	9.2182	521.1988	Isolariciresinol 4-O-glucopyranoside	1445.82	977.146	1255.76	757.242	740.472	845.633	1166.3	1222.33	1068.46	962.635	952.014	1081.05	1161.66	1156.15	1128.7	816.177	1112.44	1219.66
M479T564	9.3976	479.0823	Myricetin 3-galactoside	30.2813	13.0547	45.9828	51.4791	40.2822	50.2771	17.3638	5.24909	11.3	4.72199	14.5895	4.36223	8.57279	9.17327	3.87718	187.567	328.353	335.654
M625T572	9.5304	625.14	Myricetin 3-robinobioside	6.46328	15.8753	9.12416	9.85	10.8537	8.8559	0	0	0	0	0	0	0	0	0	38.1582	113.431	67.5927
M419T631	10.5154	419.0973	Cyanidin-3-O-alpha-arabinoside	90.9969	89.0905	93.9664	89.6887	90.5265	94.5971	88.8306	89.4651	89.2038	85.4493	88.8378	90.4967	86.6498	89.631	89.8187	89.5268	89.9329	87.2149
M773T639	10.651	773.2121	Quercetin-dihexoside-rhamnoside-isomer	34.565	32.2988	31.2781	35.8775	36.1495	49.3726	30.7491	34.874	33.5746	28.3669	33.6974	33.8198	62.9586	81.7965	69.347	43.06	55.8488	51.2681
M771T639	10.6564	771.1975	Flavonol base-4-O-hexosyl-deoxyhexosyl-hexosyl	233.014	166.728	188.676	143.405	138.37	134.633	63.4034	90.8863	51	0	0	0	432.501	461.249	311.944	138.151	190.657	205.435
M1153T651	10.8559	1153.258	Procyanidin tetramer	2161.76	2795.24	3665.44	3828.22	9406.23	5080	5544.27	6187.54	6903.8	2060.58	2943.21	3145.75	1235.5	2084.87	2194.54	491.819	2267.15	2894.6
M625T659	10.9816	625.1394	Quercetin-type flavonol dioside QDS-1	892.184	405.152	608.816	610.313	861.658	587.396	442.573	363.734	390.56	505.516	443.752	494.414	975.955	1019.21	918.427	590.534	772.803	1023.54
M335T662	11.031	335.0759	Dattelic acid	2002.92	1045.3	1631.76	851.091	1445.23	1037.84	329.857	347.363	298.044	952.648	536.478	1080.75	819.034	613.116	889.009	1319.22	2038.15	2207.23
M449T666	11.0973	449.0717	Myricetin-3-O-pentoside	47.9645	26.3193	43.1956	81.4823	66.5483	70.6712	30.1402	47.389	94.3966	9.32414	74.3879	68.5878	80.9466	109.244	45.6933	161.167	393.965	420.218
M865T667	11.1118	865.1946	Procyanidin trimer	5835.81	6334.21	10677.3	3161.07	6818.09	3191.08	15013.3	6953.22	10808.5	5836.46	10996	17243.6	5046.6	10624.4	4638.79	1031.64	4701.09	3196.01
M305T671	11.1826	305.0651	(+)-Taxifolin	88.58	94.5894	81.2912	361.091	368.673	307.374	183.569	175.617	154.793	122.62	168.893	162.776	183.425	161.245	138.761	171.918	217.469	235.155
M391T673	11.2178	391.0786	Ethyl 3-(2,3-dihydrobenzo[b][1,4]dioxin-6-yl)-7-hydroxy-4-oxo-4H-chromene-2-carboxylate	73.4077	81.6327	79.4508	94.3885	91.9983	88.3913	76.4653	76.0825	79.1495	79.4961	73.2328	74.2239	79.2269	79.075	79.0747	82.256	92.4928	83.6167
M863T673	11.2207	863.1641	Cinnamtannin B-1	48618.3	49357.4	57188.4	28809.1	45576.5	43063.7	46415.3	77042.4	78630.8	65313.8	106570	52351.8	30777.9	45579.4	43140.1	4824.29	17264.4	21615
M577T683	11.382	577.114	Procyanidin B1	4131.73	3879.7	3746.24	3016.07	5569.29	2984.8	2956.16	3749.52	3957.15	3838.08	3911.6	4491.14	3405.03	4410.52	3881.37	1579.7	2392.5	2505.75
M575T684	11.3935	575.117	Proanthocyanidin A2	275327	510408	280443	319160	232379	194951	351693	453618	391393	478034	294818	272473	200356	271252	267743	72732.1	168784	201914
M398T685	11.4139	398.0774	Tanshinone IIA sodium sulfonate	202.088	159.961	188.353	1105.62	862.874	852.956	81.9923	83.6011	82.5567	88.3905	82.9239	101.045	82.4092	93.3433	85.6819	258.648	279.056	359.618
M595T685	11.4155	595.1285	Quercetin-type flavonol dioside QDS-2	15.924	32.5896	45.8394	90.6936	94.5739	71.7716	33.853	51.3353	41.5971	13.0763	74.0992	21.038	396.359	488.031	382.569	105.981	462.993	97.4786
M611T687	11.4427	611.1359	Delphinidin-3-O-(6"-p-coumarylglucoside)	108.455	103.075	95.5944	103.049	221.52	112.176	82.448	82.3556	78.258	67.4232	68.1845	73.316	70.025	76.0621	69.0474	273.577	169.684	164.659
M555T687	11.4449	555.1705	7-O-Methylaloeresin A	97.4195	72.5676	82.4493	94.4855	97.2446	82.9185	145.033	142.874	96.0491	77.1437	111.088	136.618	101.384	105.627	92.9059	83.125	86.6271	106.78
M863T688	11.4672	863.1797	Cinnamtannin D1	10915.2	12231	9544.25	8145.19	13639.3	9506.38	9479.51	12431.2	10499.5	10674.9	18114.3	12200.1	7853.86	4810.19	6832	1037.05	3937.31	4043.04
M163T690	11.5027	163.0702	Methyl cinnamate	528.254	601.775	591.105	3359.24	3734.51	2781.2	488.285	491.265	617.299	3775.66	2216.65	2309.66	640.42	616.191	602.143	1037.01	947.502	741.561
M303T698	11.6369	303.0505	Dihydroquercetin	67.9229	20.7707	91.8441	60.025	240.701	91.4961	304.909	261.382	218.688	75.5039	50.485	50.7257	20.3483	33.6865	24.0089	34.1716	36.4729	50.861
M285T698	11.6392	285.0398	Luteolin	121.779	51.7037	78.4373	58.2138	144.987	22.9676	197.936	157.456	99.3408	41.5733	55.2197	59.3139	74.0913	72.831	42.5115	47.9219	22.4829	43.2498
M441T700	11.6733	441.1753	Lusitanicoside	5551.14	6323.15	7529.47	4752.67	9296.61	5488.04	4134.85	4376.25	3754.67	4899.05	6133.3	5234.06	4953.96	4489.68	5844.57	3692.11	4539.53	4540.82
M449T704	11.7365	449.1075	Cyanidin-3-o-beta-glucopyranoside	28400.7	28454.7	38359.9	50211.3	41091.1	35118.6	63113.5	52017.5	48869.5	37705.4	36393.2	49311.7	12850.9	7100.95	7891.98	27926.8	41250.3	37227.4
M287T704	11.7403	287.0545	Dihydrokaempferol	10292.5	8490.81	14221.8	24934.2	22590.7	15570.8	31612.7	24899	21920.8	18343.7	20201.9	26289.7	61.6684	6960.87	67.9176	10870.4	20258.1	21408.6
M610T705	11.7441	610.1474	Delphinidin 3-rutinoside	34723.9	17524.9	27003.1	17574	16680.9	17841.1	2568.5	1350.72	1130.04	7017.74	7776.69	15128.3	56159.9	36637.3	48357.2	27157.4	52275.6	87593.3
M609T705	11.748	609.1436	Quercetin-3-O-rutinoside	126147	95978.8	100881	93657.8	60042.7	106781	10030.4	6607.9	4948.35	41159.6	75946.8	55028.6	272150	224902	213059	103246	233535	198879
M611T705	11.7503	611.1588	Delphinidin-3-rutinoside	469.962	254.408	400.11	7206.89	16791.4	6817.06	229.33	244.791	178.96	1828.48	1316.38	1227.62	4065.32	2127.31	2805.43	4451.82	7439.86	6208.38
M451T706	11.7666	451.1216	(2S)-2-(3,4-dihydroxyphenyl)-5-hydroxy-7-(β-D-glucopyranosyloxy)-2,3-dihydrochromen-4-one	375.318	294.703	431.709	6295.52	10094.9	3855.84	501.138	711.659	708.211	1307.66	1122.85	978.013	315.933	385.301	344.383	1099.92	1293.24	1310.95
M579T709	11.8169	579.1388	Cyanidine-3-O-sambubioside	609	1226.57	601.677	654.225	202.931	258.229	257.794	290.455	316.012	30.617	1817.68	2398.02	442.384	885.755	914.382	245	487.15	249.596

Peak name	Retention time (min)	Mass charge ratio （Da）	Qualitative results	SB-JY			SB-GT			CP-YX			CP-GT			WHJ-JO			WHJ-LC		
				SB-1	SB-2	SB-3	SB-4	SB-5	SB-6	CP-1	CP-2	CP-3	CP-4	CP-5	CP-6	WHJ-1	WHJ-2	WHJ-3	WHJ-4	WHJ-5	WHJ-6
M199T709	11.8217	199.0971	Harmalol	2495.42	2575.2	3443.31	1456.33	1782.6	758.007	1120.55	941.283	901.22	2642.94	2390.52	2193.18	2927.58	2960.74	2106.58	963.511	1789.71	1940.06
M577T709	11.8217	577.1327	Procyanidin B3	5147.9	5107.13	5375.42	5835.15	8662.25	4701.5	2120.22	2040.74	6955.03	5004.1	5521.02	4583.65	2230.64	2819.4	1739.01	959.668	2498.64	2614.14
M401T710	11.827	401.1041	5-Hydroxyflavone	308.15	243.774	370.421	487.968	542.549	423.148	124.708	116.064	100.406	183.718	152.434	177.736	147.152	135.523	146.843	434.754	524.704	474.264
M595T710	11.8328	595.1284	Quercetin-3-O-galactoside	112839	80163.5	102516	72427.7	76229.7	48736.5	88810.2	79335.5	104183	92062.6	83188.5	78504.4	136790	101085	116602	65576.4	105443	119961
M597T711	11.8458	597.1404	Delphinidin-3-O-sambubioside	464.96	391.838	345.327	2675.78	2679.16	2207.72	214.084	337.952	261.695	805.576	365.79	575.727	516.235	562.349	245.706	440.205	1029.34	707.916
M533T713	11.8863	533.1858	phenoxy glycoside PPG-1	3624.05	3999.79	4640	1601.79	2264.04	2072.73	6304.56	5509.71	6757.38	3912.5	2599.85	3532.38	2521.37	1055.37	1937.72	746.281	784.293	1314
M464T713	11.8875	464.09	Quercetin-3-O-β-glucopyranoside	2895.28	1485.41	1845.89	3957.3	5195.47	4071.67	620.11	563.772	746.999	890.228	874.092	1109.39	6455.74	4625.06	5292.33	9213.53	12145.2	8440.24
M487T713	11.8875	487.1806	Biotin	1384.2	1124.63	1149.51	584.846	1142.64	944.964	2640.15	1873.03	2369.46	1615.81	1401.26	1543.61	1090.29	456.859	856.07	519.857	132.028	245.982
M463T713	11.8912	463.0864	Isoquercetrin	17640.2	11606.6	17668.8	34709.8	46662.6	47204.1	2872.34	2414.61	2682.48	5484.12	5531.99	6414.62	70827.5	43072.9	65143.7	76863.2	126752	100192
M575T714	11.8945	575.1174	Orotidine	221289	200483	147192	118878	142433	120274	127143	135614	133981	209667	35651.1	179884	84129.8	80740.4	85075.4	29118.7	45758.9	48131.4
M449T717	11.9436	449.1772	Dichotosin	392.413	357.869	396.712	876.977	1208.69	728.195	159.193	184.075	201.932	469.466	335.674	386.525	161.632	155.495	172.569	387.286	859.319	829.851
M247T726	12.098	247.1272	Desmotroposantonin	670.103	456.688	473.803	3304	5551.28	3125.89	534.888	548.375	481.021	3199.42	2160.3	2111.12	465.494	395.505	333.107	1120.93	1401.81	1272.44
M611T728	12.1387	611.1388	Helichrysoside	190.48	137.321	150.183	315.17	652.544	393.61	157.104	171.437	115.753	336.1	269.934	221.431	165.247	176.037	144.34	339.695	239.598	348.101
M579T730	12.1674	579.1342	Kaempferol-3-Rutinoside	2853.69	2268.05	4030.27	1826.41	2174	1841.92	2535.49	2684	2064.73	1711.81	1520.52	1713.99	4508.66	3733.06	2899.76	1101.36	2510	1719.46
M623T732	12.195	623.1595	Isorhamnetin-3-O-rutinoside	2281.65	1084.63	1629.22	257.908	399.823	146.5	1235.56	866.753	994.598	0	0	0	0	0	0	0	0	0
M625T732	12.2065	625.175	Narcissin	129.24	98.9275	103.707	179.086	173.564	121.114	91.1696	110.67	98.6475	165.257	94.173	110.514	132.006	172.098	155.68	108.673	141.571	168.629
M433T734	12.2361	433.0775	Quercetin-3-O-β-D-xylopyranoside	170137	83932.3	183750	188500	233163	161959	81220.5	83413.4	57847.2	98890.4	111649	92276.5	32053.2	330975	36735.4	523423	533529	557270
M305T735	12.2561	305.0557	Thymidine 3,5-cyclic monophosphate	321.04	198.481	306.874	816.948	1472.77	993.876	320.885	293.002	258.991	564.125	552.17	520.815	591.78	695.52	538.973	1517.26	1825.35	2246.34
M448T739	12.3164	448.0953	Rhodionin	12248.3	7632.79	9572.99	10878.1	11860.2	12979.7	12712.7	22031.2	12720.2	7364.02	6786.31	5827.81	21786.1	25637.9	17087.8	13678.9	43407.9	39169
M447T739	12.3204	447.0914	Quercetin-3-O-deoxyhexoside	118011	112699	149845	127654	159637	97131.4	154248	158232	136621	94774.7	68045.7	84098.5	222894	185872	222272	176606	261477	297031
M273T741	12.3491	273.0749	Naringerin	22443.7	24851	22968.4	131124	193977	159550	70085.5	64273.9	43113	155143	101365	124510	34905.8	30367.6	18908.5	75553.1	91851.8	113247
M505T743	12.3883	505.0968	Quercetin 3-(6''-acetylglucoside)	9175.42	3357.17	5454.97	8375.19	10252.5	12163.6	407.854	559.956	690.553	5353.38	3595.08	6824.49	16654	16921.4	18603.2	26244.4	38536.4	66514.1
M449T747	12.4469	449.1061	Taxifolin-3-O-rhamnoside	2522.24	1928.86	2582.03	1919.26	3087.16	2098.76	5146.68	5032.44	5458.01	1613.86	1342.95	1262.06	2043.78	2104.11	2174.34	1529.13	2366.25	2882.07
M435T755	12.59	435.1287	Phlorizine	302.427	290.562	323.126	151.698	290.748	186.887	360.645	368.479	372.12	301.142	273.735	295.216	217.287	251.121	192.751	131.633	278.835	212.195
M349T758	12.6279	349.1248	Riboflavin reduced	130.031	140.624	214.74	469.631	691.309	582.049	96.9474	107.456	130.44	117.489	217.63	218.794	108.685	139.844	151.717	137.419	201.88	251.413
M431T763	12.7192	431.0967	Cyanidin-3-O-rhamnoside	3079.76	1829.75	2113.97	790.484	1203.62	727.469	1843.34	1241.36	1112.77	429.126	521.471	807.067	4008.62	3609.59	3128.35	976.329	1571.17	2531.62
M303T765	12.752	303.0405	Morin	326.175	280.836	278.059	491.565	546.97	425.8	116.307	112.684	128.122	117.25	184.074	210.73	126.465	131.684	125.637	488.752	426.238	647.204
M433T766	12.7604	433.1132	4,5-Dihydroxy-2-(hydroxymethyl)-10-oxo-9,10-dihydro-9-anthracenyl hexopyranoside	190.745	267.892	148.439	330.837	408.631	288.059	72.3097	339.83	312.222	348.024	140.688	110.408	81.328	89.9108	57.9938	230.516	78.8656	360.376
M521T769	12.8186	521.1292	Anthocyanidin base-3-O-malonylhexosyl	162.046	121.544	121.465	1245.45	1500.13	800.515	137.568	132.67	125.351	371.573	270.681	280.3	140.765	110.551	116.142	260.654	295.416	289.753
M451T784	13.0734	451.1353	Catechin 7-glucoside	75.9922	111.185	137.603	30.7638	41.9061	24.8935	134.526	124.016	66.5223	364.828	290.382	342.211	64.0003	132.436	54.8991	135.411	343.895	307.353
M301T788	13.1292	301.0346	Quercetin	6.3826	6	5.36531	4	8.55129	4.13125	0	0	0	6.48491	19.1687	12	0	0	0	13.8896	6.88914	6.13017
M148T790	13.163	148.0522	E-Cinnamic acid	864.289	579.323	1115.87	323.629	409.798	327.932	956.947	798.374	694.221	723.163	738.291	792.143	1247.38	1459.91	1239.56	811.422	803.552	764.757
M163T790	13.1705	163.0756	2-Phenylethyl acetate	1375.51	1456.72	1474.05	635.415	923.904	542.533	1498.75	977.846	1265.56	1111.54	1285.5	1156.46	2157.85	2366.73	1820.41	1076.32	1080.19	1406.81
M153T795	13.2542	153.0914	Dopamine	649.676	829.226	766.029	714.119	677.684	425.883	205.152	203.401	205.674	484.7	550.711	529.14	0	24.7246	25.6538	327.802	313.089	410.53
M247T795	13.2575	247.1232	Leucodin	255.091	248.188	213.74	1640.87	2107.17	1542.62	200.366	193.667	190.882	924.95	781.765	745.513	247.171	226.458	193.078	553.109	600.656	554.5
M551T796	13.2589	551.2611	(3aR,6R,7aR)-6-methyl-3-methylidene-6-(4-oxopentyl)-3a,4,7,7a-tetrahydro-1-benzofuran-2,5-dione	94.0176	93.6638	95.4974	96.29	111.972	88.4053	88.5665	87.6864	90.4535	92.3359	89.9039	111.005	89.2923	86.7271	85.1813	90.5087	129.629	126.268
M351T802	13.371	351.1071	[4-acetyloxy-2,5-dihydroxy-6-(hydroxymethyl)oxan-3-yl] (E)-3-(4-hydroxyphenyl)prop-2-enoate	605.782	493.15	681.533	3502.62	4713.27	3146.22	1914.85	1743.96	1718.34	11118.2	7061.09	6937.03	1164.44	934.363	770.887	1246.6	1547.05	1751.19
M301T803	13.3778	301.0341	Delphinidin	1695.74	1978.94	2596.76	225.47	660.87	300.209	94.8953	116.236	107.817	127.116	157.399	121.061	2256.66	2369.27	2378.09	308.513	684.722	946.348
M129T817	13.623	129.0694	Benzylideneacetone	945.341	921.025	925.278	12336.5	4142.54	9446.85	543.919	508.5	479.624	2138.54	1035.27	1027.57	529.029	590.007	531.908	4737.91	3061.11	2531.37
M296T821	13.6782	296.1649	Hydroxyferuloylcholine	88.6533	86.02	71.4455	485.322	233.75	247.579	86.1964	73.5742	68.4466	104.571	94.5967	92.1006	94.6991	74.7742	82.2042	202.085	181.722	190.162
M223T831	13.8561	223.0399	1-Hydroxyanthraquinone	315.153	246.496	312.844	302.058	245.739	291.161	440.261	382.72	386.216	350.966	250.049	333.575	226.959	335.971	158.217	264.071	170.857	125.668
M279T834	13.9023	279.0869	6-ethyl-2,3,5-trimethyl-7H-furo[3,2-g] chromen-7-one	418.958	445.66	321.252	1594.45	2214.96	2129.19	310.375	274.604	299.147	1192.08	1726.9	1058.01	714.144	840.499	436.699	2432.43	5788.09	7035.06
M609T840	13.9935	609.124	Rutin	81.4837	83.9822	71.0002	37.2168	26.8857	42.2939	12.3997	10.5002	5.03762	62.4485	38.9937	35.1152	74.0552	45.8402	48.5592	98.9841	273.431	230.013
M838T848	14.1331	838.274	Smiglaside C	85.144	82.906	85.4431	92.4217	109.361	97.0099	84.8684	85.9595	84.604	105.948	115.645	105.702	84.9224	85.8355	86.0259	86.8503	93.6381	83.555
M369T849	14.1422	369.084	2-Deoxy-4-O-[(2E)-3-(4-hydroxyphenyl)-2-propenoyl]-3-C-(methoxycarbonyl)pentaric acid	466.825	388.429	369.232	3105.75	3655.04	1692.26	101.269	65.9443	98.3761	567.406	386.31	360.148	87.6145	85.5506	81.0721	512.374	891.381	1014.16
M193T859	14.3234	193.0864	Orthothymotinic Acid	544.597	615.966	610.77	527.792	573.207	595.253	776.679	547.879	561.199	460.806	493.294	642.382	619.429	735.384	764.497	553.112	688.606	752.778
M519T860	14.3343	519.3308	Bellericagenin B	576.385	527.874	565.08	347.271	360.264	333.224	459.063	435.771	417.585	412.849	364.329	346.692	212.968	153.976	144.728	29.7067	48.5784	43.9474
M503T877	14.6091	503.3357	Madecassic Acid	3355.98	2735.53	7953.57	2979.28	6693.06	5962.99	20327.4	15980.9	16357.4	5124.63	5525.6	6566.64	3315.45	3442.79	2580.73	216.443	292.249	321.599
M519T887	14.7809	519.3305	Cucurbitacin F	70.8243	85.7267	108.008	178.123	266.031	170.631	90.8397	162.384	137.537	126.452	141.649	96.3518	82.1241	108.442	72.9167	68.7536	67.2831	71.925
M223T907	15.1201	223.0657	Sinapoyl malate	116.402	140.53	118.014	1176.21	1115.27	906.733	132.892	122.291	132.393	379.049	296.453	271.323	140.431	153.787	141.501	533.026	339.364	393.456
M316T925	15.4175	316.2838	Dehydrophytosphingosine	1980.89	1543.39	2159.72	21482.8	28793.2	17168.3	5581.75	5716.27	5220.38	30119	29524.7	23152	3878.85	4165.18	3461.63	3603.93	5218.25	7546.23
M293T940	15.6606	293.1747	Norvaline	10481	5230.93	7034.37	9203.33	6664.89	7199.52	8663.18	6812.1	6567.37	14390.1	9734.72	11414.1	7476.37	7085.03	5476.36	9245.07	6073.34	4990.16

Peak name	Retention time (min)	Mass charge ratio （Da）	Qualitative results	SB-JY			SB-GT			CP-YX			CP-GT			WHJ-JO			WHJ-LC		
				SB-1	SB-2	SB-3	SB-4	SB-5	SB-6	CP-1	CP-2	CP-3	CP-4	CP-5	CP-6	WHJ-1	WHJ-2	WHJ-3	WHJ-4	WHJ-5	WHJ-6
M302T941	15.6817	302.3043	L-Dihydrosphingosine	19573.8	11174.3	5786.09	132004	65805.2	45702.1	9373.32	2891.33	1531.94	36720.9	16187.7	21242.2	11005.9	3740.72	1394.07	35321.2	17732.1	42402.1
M318T943	15.7141	318.2993	4-Hydroxysphinganine	12243.9	13349.1	13332.2	58108.3	66343.9	46761.4	22055.7	21115.9	20077.3	70086.9	61175.3	56906.3	20030.3	18763.9	17301.3	19097.1	29257.9	28709.3
M487T949	15.8123	487.3407	Thr Leu Lys Lys	78398.1	53718.5	76233.3	44687.9	43959.8	36657.5	43386.6	49005.9	45806.1	63389.8	51333.2	53804.1	52101.7	51862.3	35720.5	22592	24484.1	25194.7
M471T949	15.8197	471.3445	Gypsogenin	203.38	167.724	224.133	603.001	986.076	800.997	252.57	247.484	189.4	549.255	398.167	344.873	273.962	273.492	222.226	212.743	239.932	260.868
M432T978	16.3007	432.2397	Terpenoid Diterpene-1	219.597	169.595	126.308	5030.75	2398.29	1862.64	191.102	185.467	133.045	259.851	251.558	250.65	160.499	141.714	119.287	190.895	467.867	536.723
M194T978	16.3033	194.0811	6-Hydroxymethyl-7,8-Dihydropterin	17755.6	35574.3	29209.9	31182.7	22857.3	28280.6	56595.6	40485.5	44719.7	52088.5	24128.8	20235.2	26542.7	26847.2	30853.1	27828.8	26918.3	43119.2
M251T985	16.4242	251.1638	Ngaione	415.625	274.26	302.525	4526.46	1583.66	1644.68	379.003	336.196	316.43	824.294	679.012	632.916	280.502	288.764	290.765	766.903	1134.76	1021.16
M452T1044	17.3983	452.2767	Lyso-Phosphatidylethanolamine 16	12330.1	5454.43	5483.19	2199.38	4723.79	2502.82	2215.19	2176.2	2620.03	2333.62	2044.45	2246.52	685.92	651.318	716.472	1064.92	1078.99	2755.6
M496T1051	17.524	496.3388	1-Palmitoyl-sn-glycero-3-phosphocho line	5201.98	3349.03	3882.59	19600.8	24564.1	18993.4	2724.44	2584.01	2632.07	30490.4	28315.5	26686.7	6213.08	6788.31	4657.76	5312.57	9172.53	11018.1
M540T1052	17.525	540.3288	Hexadecanoyl-hydroxy-sn-glyceropho sphocholine (PUT)	19896	16943.4	27214	11968.9	14303.8	8203.24	9377.74	4156.1	4065	20889.4	16831.5	23326.3	3413.91	2059.86	2818.57	3280.82	7082.87	14427.2
M279T1054	17.566	279.2314	9(R)-HODE	136.37	145.791	146.663	670.037	466.873	431.541	113.948	105.16	120.24	280.031	188.512	192.963	117.475	118.117	142.612	227.818	211.389	218.413
M295T1055	17.5763	295.2269	(±)-12(13)-epoxy-9(Z)-octadecenoic acid	309.465	544.347	673.684	324.748	479.73	287.761	227.997	182.742	133.177	399.956	492.336	317.248	282.588	211.895	271.657	365.057	252.342	217.081
M356T1057	17.6092	356.0791	Dihydrosanguinarine	117.298	108.546	87.6453	186.703	247.462	182.964	108.77	110.225	96.027	5823.11	4480.89	4350.15	103.399	105.698	95.937	459.209	441.294	545.182
M522T1069	17.8147	522.3547	1-Oleoyl-sn-glycero-3-phosphocholin e	558.166	386.937	420.348	1151.98	1658.3	1097.41	383.544	260.676	266.771	2136.96	1461.53	1341.31	189.266	240.41	207.513	608.752	766.56	782.221
M783T1095	18.2438	782.5672	1-Hexadecanoyl-2-(9Z-octadecenoyl)-sn-glycero-3-phosphocholine	487.65	418.088	397.91	2970.19	3060.18	1573.32	763.809	644.378	636.696	1057.16	875.711	685.779	773.036	905.568	592.013	824.806	503.305	422.542
M759T1095	18.2442	758.5667	1-Hexadecanoyl-2-octadecadienoyl-sn -glycero-3-phosphocholine	414	556.954	606.715	2402.6	3285.13	3413.18	858.355	160.634	1142.8	2227.97	2214.58	2129.26	560.823	888.788	977.25	1043.1	1057.39	1149.98
M279T1101	18.3447	279.1525	Pantetheine	1962.76	1832.94	2072.75	148419	61116.2	40697.1	1808.61	1615.07	1957.82	5145.28	5245.78	3967.67	1878.61	1974.4	1839.05	7313.08	7891.69	7779.54
M524T1128	18.8033	524.3703	1-Stearoyl-2-hydroxy-sn-glycero-3-ph osphocholine	336.555	298.577	332.415	772.198	776.171	919.081	187.92	177.702	165.818	1182.93	1002.9	677.774	234.477	244.53	245.853	250.547	359.865	399.432
M507T1151	19.1888	507.2736	Asn Phe Ala Arg	117.526	400.768	146.44	209.981	167.035	204.087	1731.82	1468.24	530.155	917.165	516.921	760.761	229.966	245.337	264.852	429.388	172.148	639.755
M277T1163	19.3864	277.2162	α-Eleostearic Acid	4457.94	3179.02	4352.92	1964.57	3469.41	2232.92	826.723	847.542	732.699	2795.7	3032.82	2973.49	1996.11	2761.14	2276.25	618.501	1066.48	1411.78
M699T1174	19.5726	698.5539	Galactosylceramide	375.036	406.356	353.033	861.681	893.023	1171.03	465.57	350.04	351.921	1029.44	686.388	737.733	464.685	479.442	411.671	1086.67	321.53	466.85
M439T1188	19.8046	439.3562	Ursolic acid	1953.03	1531.85	1823.43	7311.86	14506.3	14425.5	3761.69	4486.03	3837.18	13212.8	11524.9	8677.67	5666.12	6374.95	5167.4	6697.74	8424.24	9738.77
M787T1189	19.8147	786.5947	1,2-Dioleoyl-Phosphatidylcholine	260.928	320.803	683.313	683.012	1248.58	1243.09	405.486	384.501	303.043	446.861	429.779	398.944	189.152	212.282	254.218	352.631	472.802	492.248
M531T1198	19.9683	531.2749	Pro Ala Asp Lys Thr	376.423	456.246	538.868	4295.53	2730.66	1028.98	638.318	568.974	275.357	93.7456	102.452	277.7	378.377	479.572	366.09	93.7218	388.242	3050.52
M279T1201	20.0102	279.2314	9(E),11(E)-Conjugated Linoleic Acid	25398.5	12730.1	25381.2	23892.8	19359.9	19266.4	9305.34	9123.34	9027.73	25244.3	21093.5	13097.6	19952.9	22652.2	22889.7	9279.99	8895.23	10737
M757T1212	20.201	756.5508	Phosphatidylcholine-Diacylglycerol	4041.22	4061.1	184.378	3033.73	2448.67	1885.63	306.955	3547.08	3549.19	2652.8	1569.83	1726.16	1122.64	1205.49	1076.77	1032.35	895.935	863.163
M311T1223	20.3876	311.167	1-[2-methyl-6-[(2S,3R,4S,5S,6R)-3,4,5-trihydroxy-6-(hydroxymethyl)oxan-2-yl]oxyphenyl]ethanone	13450.4	15601.1	67148	54793	105690	105181	12955.2	104343	28913.7	31200	51091.3	24923	32427.9	182456	26391.4	61016.6	42491.6	93708
M593T1227	20.4475	593.2745	Pheophorbide A	14302.5	35877.3	32157	65346.7	137762	159680	288.551	688.352	1155.22	20335.8	46401.8	21682.6	5756.16	34906.8	28533.2	4486.92	63752.5	109690
M595T1227	20.448	595.2812	Bebeerine	395.728	707.397	1235.35	1557.8	2875.48	4091.99	88.3712	106.505	120.915	338.612	801.707	407.035	174.633	636.533	727.573	223.474	1299.46	2384.12

Supplementary Table S3. The correlation coefficients of core flavor compounds in Younai fruits.

	Total soluble solids	Titratable acidity	Sugar-acid ratio	Firmness	Fructose	Glucose	Sorbitol	Malic acid	Alcohols	Aldehydes	Esters	Lactones	Terpenoids
Total soluble solids	1.00	0.10	0.84	-0.98	0.71	0.32	0.23	-0.33	0.41	-0.63	0.23	-0.04	-0.44
Titratable acidity	0.10	1.00	-0.46	-0.14	-0.13	-0.48	0.59	-0.84	-0.23	-0.18	0.01	0.36	0.62
Sugar-acid ratio	0.84	-0.46	1.00	-0.79	0.70	0.59	-0.10	0.16	0.49	-0.43	0.20	-0.27	-0.77
Firmness	-0.98	-0.14	-0.79	1.00	-0.81	-0.28	-0.16	0.29	-0.33	0.74	-0.41	-0.02	0.41
Fructose	0.71	-0.13	0.70	-0.81	1.00	0.47	-0.39	0.21	-0.05	-0.74	0.83	0.19	-0.42
Glucose	0.32	-0.48	0.59	-0.28	0.47	1.00	-0.39	0.40	-0.05	0.22	0.24	-0.21	-0.70
Sorbitol	0.23	0.59	-0.10	-0.16	-0.39	-0.39	1.00	-0.92	0.59	0.09	-0.58	-0.43	0.04
Malic acid	-0.33	0.84	0.16	0.29	0.21	0.40	-0.92	1.00	-0.31	0.09	0.33	0.11	-0.25
Alcohols	0.41	-0.23	0.49	-0.33	-0.05	-0.05	0.59	-0.31	1.00	-0.08	-0.43	-0.82	-0.62
Aldehydes	-0.63	-0.18	-0.43	0.74	-0.74	0.22	0.09	0.09	-0.08	1.00	-0.63	-0.34	-0.02
Esters	0.23	0.01	0.20	-0.41	0.83	0.24	-0.58	0.33	-0.43	-0.63	1.00	0.39	-0.07
Lactones	-0.04	0.36	-0.27	-0.02	0.19	-0.21	-0.43	0.11	-0.82	-0.34	0.39	1.00	0.74
Terpenoids	-0.44	0.62	-0.77	0.41	-0.42	-0.70	0.04	-0.25	-0.62	-0.02	-0.07	0.74	1.00

Supplementary Table S4. The *p* values of the correlation analysis of core flavor compounds in Younai fruits.

	Total soluble solids	Titratable acidity	Sugar-acid ratio	Firmness	Fructose	Glucose	Sorbitol	Malic acid	Alcohols	Aldehydes	Esters	Lactones	Terpenoids
Total soluble solids	0.0000	0.8571	0.0385	0.0007	0.1161	0.5317	0.6555	0.5276	0.4254	0.1821	0.6618	0.9425	0.3792
Titratable acidity	0.8571	0.0000	0.3543	0.7983	0.8114	0.3353	0.2150	0.0379	0.6657	0.7398	0.9895	0.4863	0.1852
Sugar-acid ratio	0.0385	0.3543	0.0000	0.0594	0.1218	0.2177	0.8447	0.7622	0.3188	0.3951	0.7021	0.6063	0.0723
Firmness	0.0007	0.7983	0.0594	0.0000	0.0514	0.5864	0.7610	0.5723	0.5186	0.0915	0.4227	0.9749	0.4204
Fructose	0.1161	0.8114	0.1218	0.0514	0.0000	0.3451	0.4460	0.6963	0.9311	0.0925	0.0430	0.7185	0.4084
Glucose	0.5317	0.3353	0.2177	0.5864	0.3451	0.0000	0.4496	0.4317	0.9266	0.6701	0.6459	0.6962	0.1204
Sorbitol	0.6555	0.2150	0.8447	0.7610	0.4460	0.4496	0.0000	0.0085	0.2181	0.8602	0.2279	0.3947	0.9388
Malic acid	0.5276	0.0379	0.7622	0.5723	0.6963	0.4317	0.0085	0.0000	0.5448	0.8588	0.5209	0.8356	0.6392
Alcohols	0.4254	0.6657	0.3188	0.5186	0.9311	0.9266	0.2181	0.5448	0.0000	0.8818	0.3952	0.0440	0.1861
Aldehydes	0.1821	0.7398	0.3951	0.0915	0.0925	0.6701	0.8602	0.8588	0.8818	0.0000	0.1772	0.5089	0.9651
Esters	0.6618	0.9895	0.7021	0.4227	0.0430	0.6459	0.2279	0.5209	0.3952	0.1772	0.0000	0.4392	0.9017
Lactones	0.9425	0.4863	0.6063	0.9749	0.7185	0.6962	0.3947	0.8356	0.0440	0.5089	0.4392	0.0000	0.0942
Terpenoids	0.3792	0.1852	0.0723	0.4204	0.4084	0.1204	0.9388	0.6392	0.1861	0.9651	0.9017	0.0942	0.0000
